# Exploring Metal-Free Click Reactions: New Frontiers in Glycochemistry and Bioconjugation

**DOI:** 10.1021/acs.bioconjchem.5c00049

**Published:** 2025-07-17

**Authors:** Pedro Ramírez-López, José Ramón Suárez, Aida Flores, María J. Hernáiz

**Affiliations:** Departamento de Química en Ciencias Farmacéuticas, 73076 Facultad de Farmacia, Universidad Complutense de Madrid, Plz. Ramón y Cajal s/n, Madrid, C.P. 28040, España

## Abstract

Efficient and biocompatible methods for synthesizing glycoconjugates are essential in chemical biology, as these molecules play pivotal roles in cellular recognition, signaling, and immune responses. Abnormal glycosylation is associated with diseases such as cancer, infections, and immune disorders, positioning glycoconjugates as promising candidates for therapeutic, diagnostic, and drug delivery applications. Traditional chemical approaches often lack biocompatibility and efficiency; however, the advent of metal-free click chemistry has revolutionized glycoconjugate synthesis by providing selective and versatile tools under mild conditions. This review highlights four remarkable metal-free click reactions: thiol–ene coupling (TEC), strain-promoted azide–alkyne cycloaddition (SPAAC), inverse electron-demand Diels–Alder (IEDDA) reaction, and sulfur fluoride exchange (SuFEx). TEC enables the regio- and stereoselective synthesis of glycoconjugates, including S-polysaccharides, glycopeptides, and glycoclusters, advancing vaccine development and carbohydrate-based therapeutics. SPAAC, a bioorthogonal and metal-free alternative, facilitates *in vivo* imaging, glycan monitoring, the synthesis of glycofullerenes and glycovaccines, and the development of targeted protein degradation systems such as lysosome-targeting chimeras (LYTACs). Additionally, the combination of SPAAC with biocatalysis offers a sustainable approach for preparing glycoconjugates with therapeutic potential. The IEDDA reaction, a highly efficient metal-free biorthogonal cycloaddition, plays a key role in metabolic glycoengineering for live-cell imaging and glycan-based therapies and also contributes to the creation of injectable hydrogels for drug delivery and tissue engineering. SuFEx, a more recent reaction, enables efficient sulfonamide and sulfonate bond formation, broadening the toolbox for glycoconjugate and protein functionalization. These methodologies are transforming glycochemistry and glycobiology, driving advancements in biomedicine, materials science, and pharmaceutical development.

## Graphical abstract

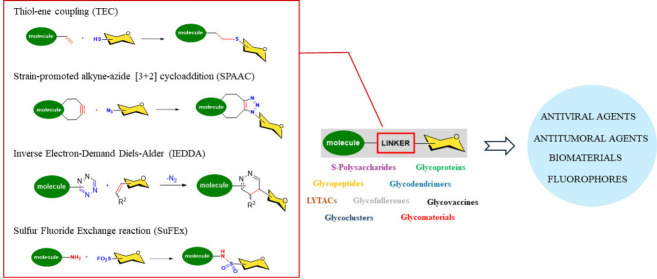

## 1 Introduction

In the dynamic field of chemical biology, developing efficient, selective, and biocompatible strategies for synthesizing glycoconjugates is a central goal in glycochemistry and glycobiology. Glycoconjugates, such as glycoproteins, glycolipids, and proteoglycans, play crucial roles in cell recognition, signaling, adhesion and immune response.^1–3^ They are also implicated in various pathological processes,^4^ including cancer,^5,6^ infections,^7–11^ inflammation, neurodegenerative disorders, and coagulation,^12^ making them valuable targets for therapeutic and diagnostic applications.^13,14^

Traditional synthesis methods are often too harsh for biological systems. However, the introduction of click chemistry by Sharpless in 2001 ^15^ revolutionized glycoconjugates synthesis. Click chemistry refers to a set of highly selective, efficient and versatile reactions that proceed under mild conditions, making it a valuable tool in carbohydrate chemistry.^16^ Among these, the Huisgen 1,3-dipolar cycloaddition between azides and alkynes has proven particularly significant in organic chemistry, offering broad utility across numerous scientific disciplines.^17–23^ This reaction is catalyzed by copper ions, which also confer high regioselectivity, and it is therefore known as the copper-catalyzed Huisgen 1,3-dipolar cycloaddition (CuAAC). However, concerns about copper’s cytotoxicity and its interference with biological processes have limited its use in biological systems.^24,25^ As a result, metal-free click reactions have gained growing interest. Some of these reactions are bioorthogonal, meaning that they can proceed inside living organisms without disrupting native biochemical processes. These features have made them essential tools for real-time biomolecule labeling, imaging, and modification.^25–27^ Indeed, recent studies highlight metal-free click chemistry as a preferred approach in the design of biocompatible glycoconjugates.^28,29^

This review focuses on four key metal-free click reactions (Table 1): thiol–ene coupling (TEC), strain-promoted azide–alkyne cycloaddition (SPAAC), inverse electron-demand Diels–Alder (IEDDA) reaction, and sulfur fluoride exchange (SuFEx). Among them, SPAAC and IEDDA are bioorthogonal, allowing selective modification of biomolecules in living systems. In contrast, TEC and SuFEx though not bioorthogonal offer complementary advantages in reactivity and synthetic versatility (Table 1).

**Table 1:**
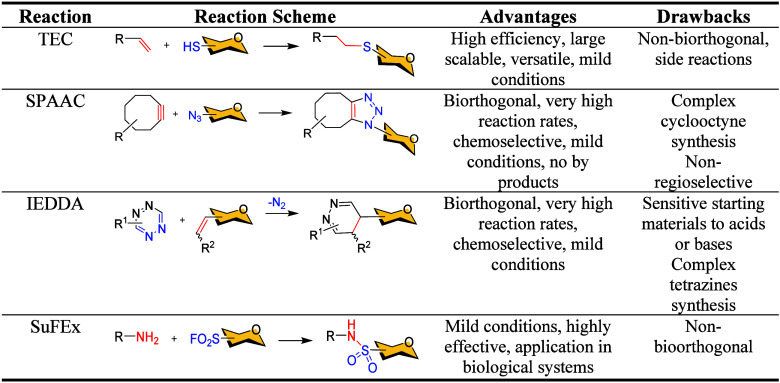
Properties of the Metal-Free Click Reactions Covered in This Review

TEC, also known as hydrothiolation, enables efficient formation of stable thioethers under mild, often aqueous, conditions.^30^ Driven by visible light via a radical mechanism, TEC is atom-economical and compatible with diverse functional groups, making it highly versatile in glycochemistry (Table 1).^31–35^

SPAAC is a metal-free [3 + 2] cycloaddition between azides and strained cyclooctynes. Developed as a bio-orthogonal alternative to CuAAC, SPAAC proceeds efficiently under physiological conditions without toxic catalysts. It offers high chemoselectivity, biocompatibility, and fast kinetics. Despite limitations such as regioisomer formation and the synthetic complexity of cyclooctynes, SPAAC remains a valuable tool in carbohydrate chemistry (Table 1).^36–38^

IEDDA reaction is a rapid and selective cycloaddition between electron-deficient dienes (e.g., tetrazines) and electron-rich dienophiles (e.g., strained alkenes). It is bioorthogonal and proceeds efficiently under mild, aqueous conditions, making it highly suitable for chemical biology applications (Table 1).^39–41^

SuFEx is a newer click reaction that forms S–O and S–N bonds through reactions between sulfonyl fluorides or fluorosulfates and nucleophiles, such as silyl ethers or amines. It is driven by strong Si–F bond formation and proceeds under mild metal-free conditions. SuFEx offers high stability, chemoselectivity, and compatibility with biological environments (Table 1).^42,43^

Together, these metal-free reactions expand the toolbox for glycoconjugate synthesis, offering new possibilities for therapeutic and diagnostic innovation. This review highlights their mechanisms and applications, underscoring their potential to advance bioconjugation and glycochemistry in both biotechnology and pharmaceutical development.

## 2 Thiol–Ene Coupling (TEC)

### 2.1 Introduction

Hydrothiolation of terminal inactivated alkenes, also known as TEC, is a century-old reaction that has emerged as an effective and rapid metal-free click reaction between thiols and alkenes to create stable thioethers.^30^ This reaction proceeds with excellent yields and complete selectivity without the need for a metal catalyst, and it is compatible with aqueous conditions. Originally discovered by Posner^44^ in 1905, this reaction is driven by visible light, which supports a free radical mechanism. The process begins with the generation of a thiyl radical from a thiol, either by direct UV irradiation (at wavelengths between 254–405 nm) or in the presence of a radical photoinitiator, which induces homolytic cleavage of the sulfhydryl S–H bond. The thiyl radical then undergoes anti-Markovnikov addition to an alkene, forming an intermediate carbon center radical. This carbon radical subsequently abstracts a hydrogen atom from a second molecule of thiol affording the thioether product and a new thiyl radical, which perpetuates the radical cycle (Scheme 1A).^45,46^

**Scheme 1:**
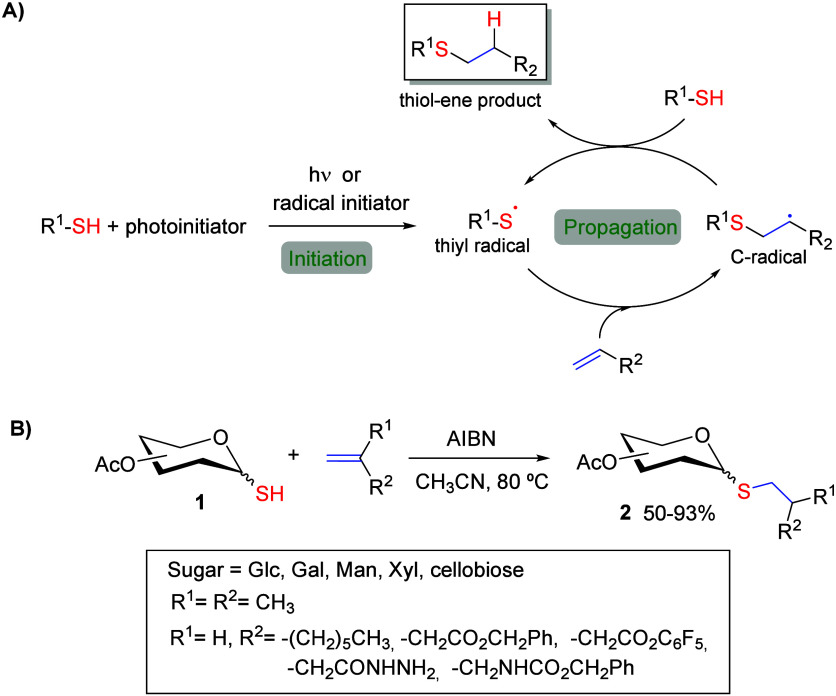
(A) Mechanism Pathway for Anti-Markovnikov Thiol–ene Reaction; (B) Thiol–Ene Reaction between Thioglycosides and Terminal Alkenes

The high atom economy and the effectiveness of the TEC reaction significantly simplify the reaction workup and isolation of the final alkyl sulfide products. However, a primary limitation of this click reaction is the reversibility of the thiyl radical addition to the alkene step, conducting in some cases to the detection of a disulfide side product, coming from the thiyl radical homocoupling. The irreversibility of the reaction can be forced by carefully designing the structure of both reactants and optimizing reaction conditions, such as the alkene-to-thiol ratio, photoinitiator concentration, solvent and pH.^30,47,48^

The exceptional versatility of thiol–ene chemistry has expanded its applicability across various fields, including polymers and surface chemistry,^31,45,46^ natural products and peptide synthesis.^32,49^ Given the numerous benefits of TEC and the ubiquity of the carbon–sulfur bond in natural products and bioconjugate chemistry, TEC offers a useful and effective assembling platform for carbohydrate chemistry.^33,35^ Specifically, thioglycosides play a significant role not only in biological function and drug design but also in synthetic chemistry as a key precursor for other glycosides, glycans, and glycoconjugates.^34^ The first thiol–ene reaction involving thioglycosides and alkenes was reported in 1988 by Pavia and co-workers.^50^ Alkyl 1-thioglycosides **2** were prepared in moderate to excellent yields by reacting peracetylated 1-thioglycoside **1** (mono- or disaccharide) with alkenes in acetonitrile at 80 °C using α-azobis­(isobutyronitrile) (AIBN) as a radical initiator (Scheme 1B). Notably, this methodology allows the synthesis of alkylated thioglycosides containing different functional groups at the terminal position of the aglycon chain (R^2^), which are suitable for further conjugation with other biomolecules. Since this pioneering work, numerous articles have been published on the synthesis of thiosugars and sulfur containing glycoconjugates via TEC.^33^

### 2.2 Applications

#### 2.2.1 Synthesis of S-Polysaccharides via TEC

Glycomimetics are synthetic compounds designed to mimic the structure and function of carbohydrates, which play crucial roles in various biological processes such as cell recognition, signaling, and immune response. These compounds are useful for studying these processes and hold the potential for developing therapeutic agents that target carbohydrate-binding proteins involved in diseases such as cancer, inflammation, and infectious diseases. The application of TEC to the synthesis of *S*-linked oligosaccharides has attracted significant attention in the carbohydrate field because *S*-linkage is more resistant to acid hydrolysis or enzymatic degradation, offering enhanced stability, bioavailability and selectivity compared to *O*-glycosidic linkage.^51^ Dondoni and co-workers first applied this efficient methodology in 2009, performing the coupling between peracetylated thiol sugars **3a**–**b** and *exo*-glycals **4** as a strategy for the synthesis of *S*-linked disaccharides **5** (Scheme 2).^52^ The reaction mixture was irradiated with a UV–visible lamp (λ_max_ = 420 nm) in the presence of 2,2-dimethoxy-2-phenylacetophenone (DPAP) as the photoinitiator. The 1,6-linked *S*-disaccharides were rapidly obtained in excellent yields with high diastereoselectivity, confirming the potential of TEC in glycochemistry. The same authors extended this methodology to evaluate the hydrothiolation reaction with the derived *endo*-glycals **6**. The reaction was completely regioselective, with the thiyl radical attacking exclusively at the C-2 position of the glycal. However, the nature of the glycal moiety determined whether the coupling product was a single stereoisomer or as a mixture of stereoisomers **7–8** (Scheme 2).^53^ Alternatively, Borbás and co-workers reported a similar study aimed at preparing new types of glycomimetic compounds with potential therapeutic applications.^54^ The addition of sugar thiol **3** to *exo*-galactal **9**, which bears a double bound at the anomeric position, or 2,3-unsaturated glycoside **11**, containing an *endo*-alkene, successfully afforded the corresponding compounds **10** and **12** respectively as single regioisomers. Remarkably, in this case, only one stereoisomer was detected (Scheme 2).

**Scheme 2:**
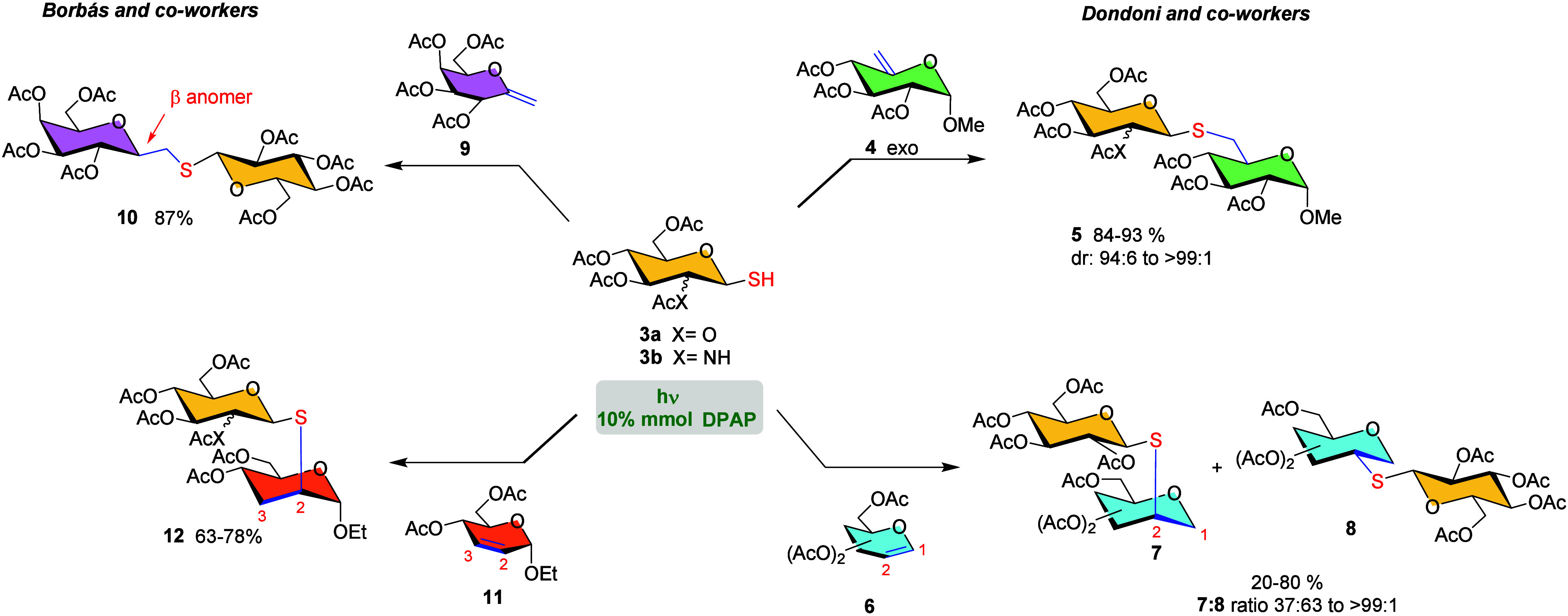
Synthesis of *S*-Linked Disaccharides *via* TEC

In 2017 Borbás and co-workers employed the same strategy for the synthesis of sugar-modified nucleosides.^55^ The therapeutic application of nucleosides and nucleic acids has promoted the development of nucleoside analogues with improved chemical and biological properties.^56^ However, achieving versatile and stereoselective alterations of the furanose residue in nucleosides to create new drug candidates remains a significant challenge for synthetic chemists. In this study, the authors demonstrated that the low temperature TEC reaction (−80 °C) between *C*3′-methylene derivatives of uridine **15a** and ribothymidine **15b** as ene-nucleosides provided an easy and efficient approach to prepare pyranose-modified nucleosides **16** (Scheme 3). Remarkably, the addition of the thioglycoside **13a**–**b** to the double bound proceeded with excellent level of d-*xylo* selectivity.

**Scheme 3:**
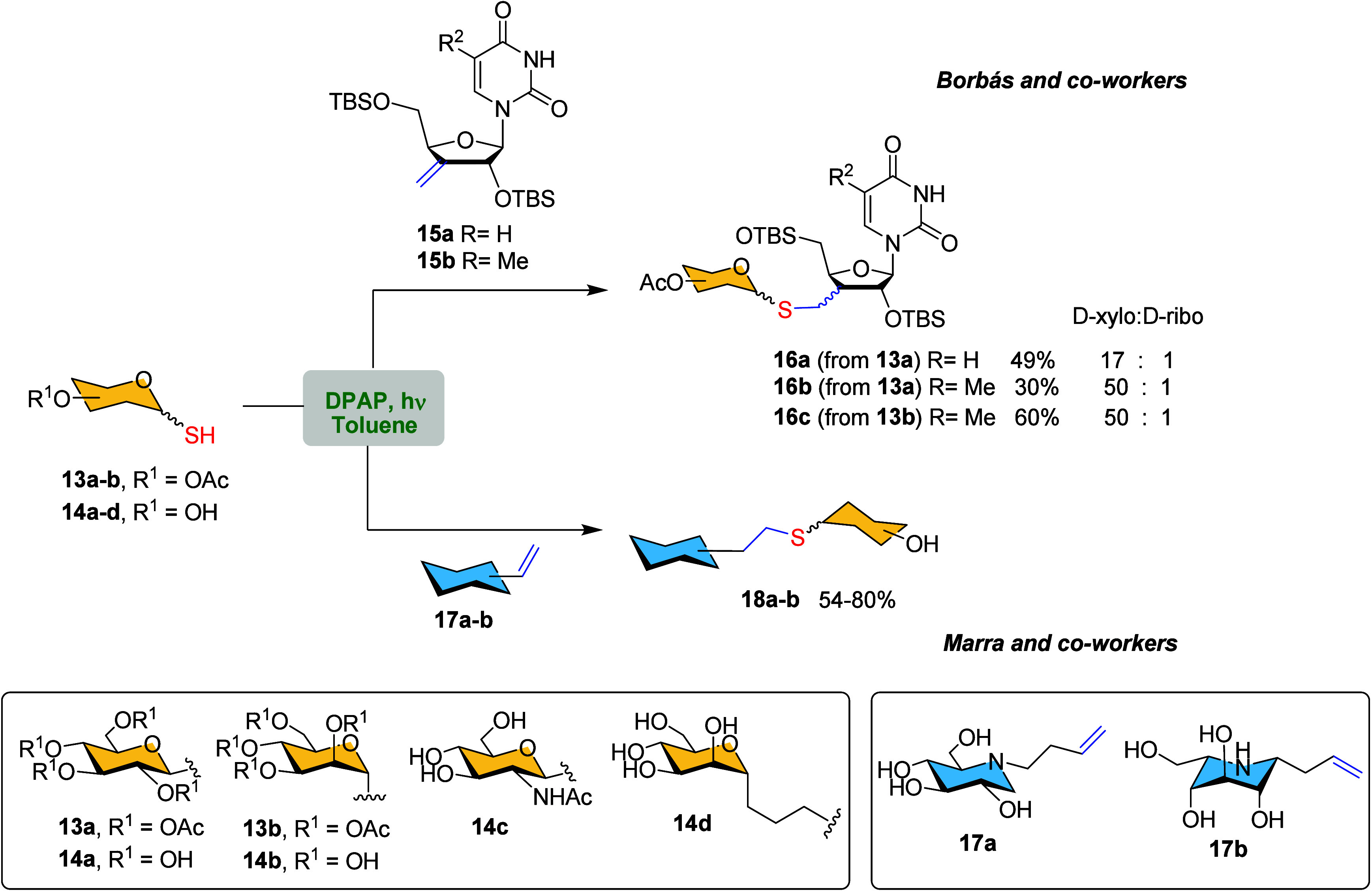
Synthesis of Sugar-Modified Nucleosides **16a**–**c** and *N*-Linked and *C*-Linked Imino-disaccharides **18a**–**b** *via* TEC^a^

a TBS = *t*-Butyldimethylsilyl.

Iminosugars are another glycomimetic compound that contain a nitrogen atom instead of an oxygen in the ring structure. They are known for their ability to inhibit glycosidase enzymes,^57^ making them valuable in various therapeutic applications, including the treatment of viral infections, diabetes, and lysosomal storage disorders. Recently, Marra et al. first reported the efficient synthesis of *N*-linked imino-disaccharides **18a** or anomerically *C*-linked imino-disaccharides **18b** *via* TEC from different sugar thiols **14a**–**d** and the corresponding iminosugar alkenes **17a** or **17b** (Scheme 3).^58^ The photoinduced radical addition reactions were performed under similar reaction conditions as previously described, with the addition of trifluoroacetic acid (TFA) to prevent the deprotonation of the sugar thiol by the iminosugar, thereby favoring the formation of a thiyl radical necessary for the coupling.

In a similar fashion, Lázár and co-workers reported the synthesis of more complex oligosaccharide homologues by photoinitiated TEC reaction with complete regio- and stereoselective control.^59^ The authors began preparing the α-*S*-linked disaccharide **21** through the free-radical addition of thiol **20** to 2-acetoxyglucal **19** in toluene at −80 °C, by irradiation at λ_max_ = 365 nm in the presence of the photoinitiator DPAP (Scheme 4). Subsequent, selective *S*-deacetylation of **21** to yield free thiol **23**, followed by conjugation with another 2-acetoxyglucal **19** monosaccharide *via* the TEC reaction, allowed the effective creation of trisaccharide **25**. Additionally, a tetrasaccharide structure was generated through the same TEC conditions to incorporate thiol disaccharide **23** into disaccharide 2-acetoxyglucal **22**, which was prepared from **21** by regenerating the double bond under basic conditions. By following similar sequential reactions, we also achieved the rapid and efficient preparation of pentasaccharide **26** was also achieved.

**Scheme 4:**
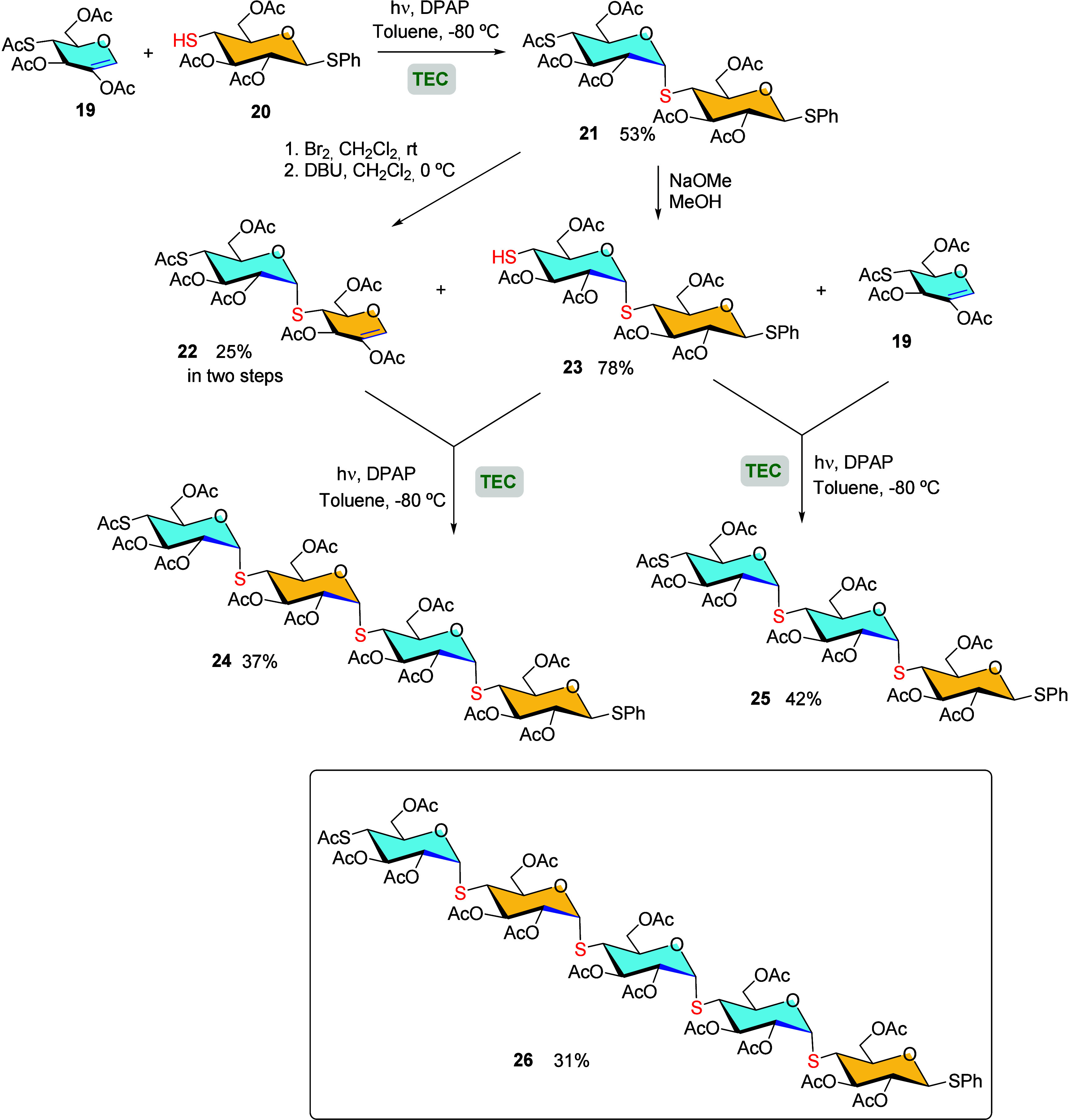
Synthesis of Oligosaccharide Homologues by TEC

#### 2.2.2 Synthesis of Sugar-Peptides/Proteins via TEC

Glycopeptides play important roles in biology and medicine, serving as indispensable tools in fundamental biological processes, where the glycan and the peptidyl substructures often exhibit distinct and complex function, such as cellular recognition, adhesion, growth and differentiation.^60^ Moreover, aberrant glycosylation is associated with autoimmune and infectious diseases as well as cancer. The challenges in isolating glycopeptides or glycoproteins from natural sources have hampered efforts to elucidate the individual biological functions of glycoproteins, particularly when the precise structure of the glycan determines biological activity. Therefore, the development of new methods for linking sugars to peptides or proteins, with a well-defined structure, is an active area of research.^61^ Due to its mild conditions and high regioselectivity, the TEC reaction has become an effective synthetic approach for sugar-peptide or protein bioconjugation.

One of the first examples reported in the literature dates back to 2001, when Klaffke and co-workers described an elegant methodology for preparing *N*-linked neoglycopeptides **31a** through a hydrotiolation reaction (Scheme 5A).^62^ The synthesis involved a photoinduced (254 nm) thiol–ene coupling of *N*-allyl glycosides **27a**–**c** (α-d-Glc, β-d-Glc, β-d-GlcNAc and β-d-Gal) with cysteamine at room temperature, introducing a terminal amino-thioether spacer, followed by cross-linking of the corresponding amino group with Cbz-Gln-Gly dipeptide, catalyzed by microbial transglutaminase (TGase) to easily access to the desired neoglycopeptides.

**Scheme 5:**
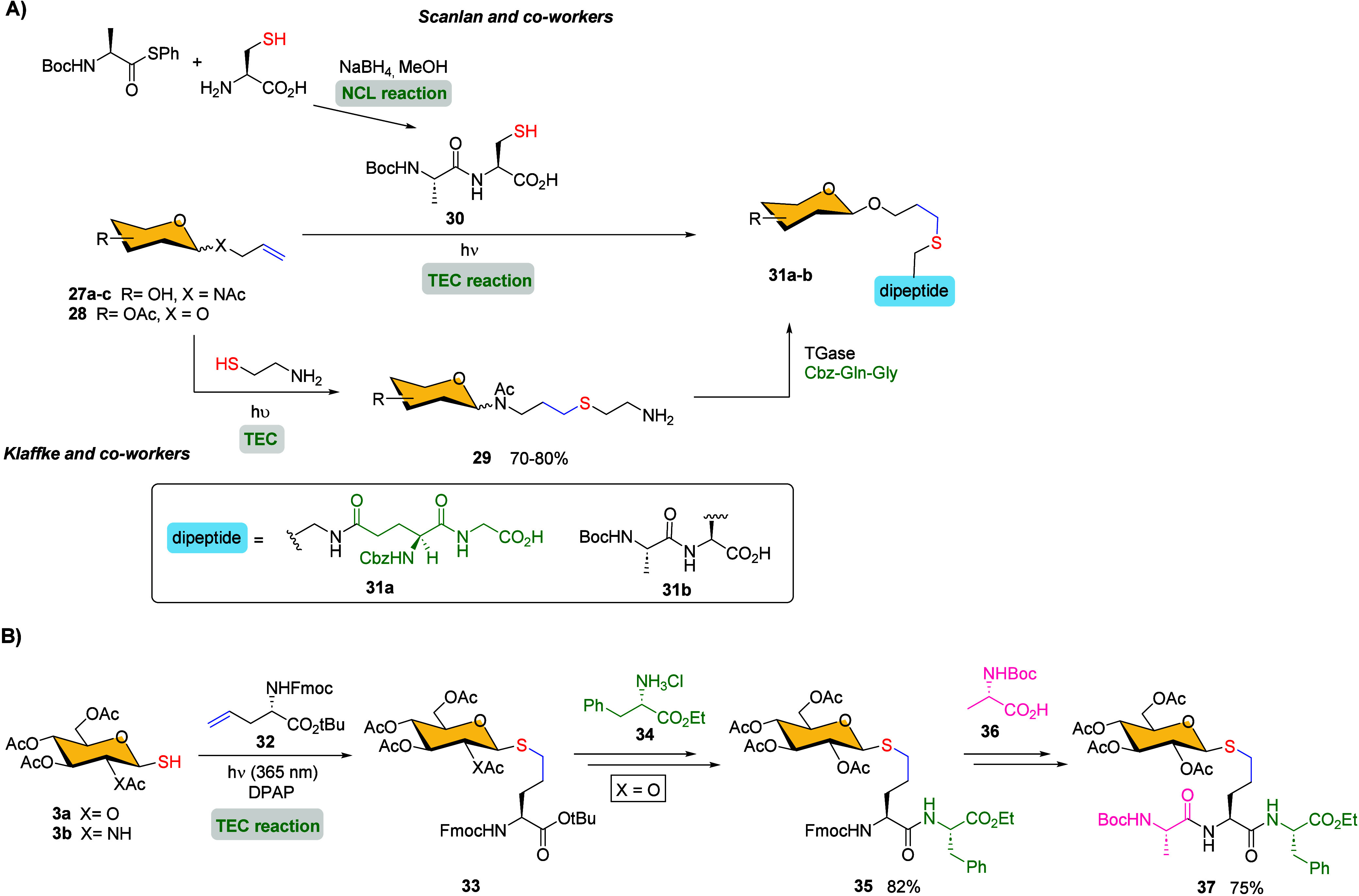
(A) Synthesis of Neoglycopeptides **31a**–**b**;^a^ (B) Synthesis of S-Glycopeptide **37** ^b^

a Boc = *t*-butyloxycarbonyl. Cbz = (phenylmethoxy)­carbonyl group. TGase = glutaminyl-peptide γ-glutamyl transferase.
b Fmoc = Fluorenylmethoxycarbonyl.

The versatility of the thiyl radical click reaction was also demonstrated by Scanlan and co-workers in a sequential combination of native chemical ligation and thiol–ene radical chemistry (NCL-TEC) as a novel methodology for rapidly accessing functionalized glycopeptides.^63^ The NCL reaction between Boc-protected alanine thioester and cysteine furnished dipeptide **30** in high yield. Then, the introduction of a galactose ring containing an anomeric terminal alkene **28** by the TEC reaction gave the desired thioether-linked compound **31b** in 87% yield (Scheme 5A). In this instance, DPAP was employed as radical initiator, and the addition of 4-methoxyacetophenone (MAP) as photosensitizer significantly increased the reaction yield.

Dondoni et al. reported a complementary strategy to prepare *S*-glycopeptide **37** *via* TEC reaction, this time using a saccharide bearing a thiol group as the starting material.^64^ The coupling reaction between glycosyl thiols **3a**–**b** and alkenyl glycine **32** by means of TEC, followed by the sequential incorporation of orthogonally protected amino acids into glycosylated TEC product **33**, successfully afforded *S*-glycopeptide **37** (Scheme 5B).

*O*-Acetyl moieties in glycosyl thiols **3** were selected as protecting groups due to their compatibility with the photoreaction conditions. In addition, orthogonal protective groups in the amino acids were employed to obtain target *S*-glycosyl tripeptide **37**, suitable for further elongation through peptide synthesis.

To simplify the synthesis process and reduce waste, Fairbanks and colleagues developed a method for preparing glycoconjugates that does not require protecting groups. Their approach is based on a combined two-click reaction process, which maintains selectivity and yield while eliminating the need for additional steps typically associated with protecting group strategies.^65^ First, the introduction of the alkene moiety into the sugar ring was achieved *via* CuAAC between the *in situ* formed glycosyl azide from aminosugar **38** and the allyl propargyl ether (Scheme 6).

**Scheme 6:**
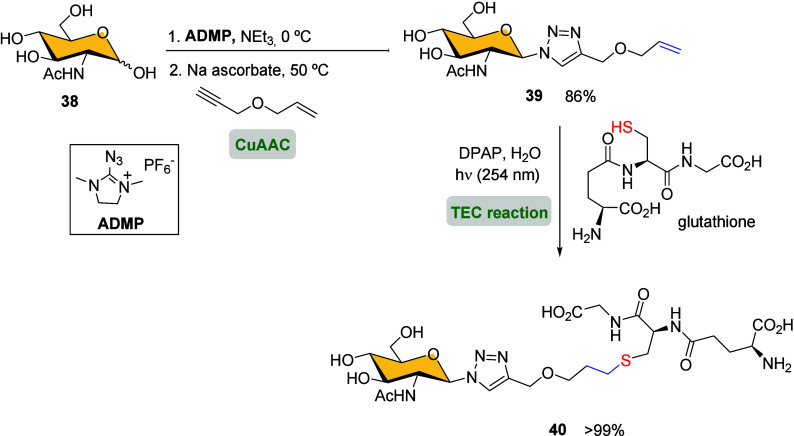
Synthesis of Glycopeptide **40** by Double-Click Approach^a^

a AMDP = 2-azido-1,3-dimethylimidazolinium hexafluorophosphate.

Then, the reaction of alkene-aminosugar **39** with glutathione afforded the corresponding glycopeptide **40** through thiol–ene click coupling in quantitative yield.

The scope of the TEC was expanded to more complex systems when Davis and co-workers first investigated the synthesis of *S*-linked glycoproteins in 2009, where site-specific ligation of glycosyl thiols to olefinic proteins was achieved.^66^ This approach utilized the incorporation of a non-natural amino acid, homoallylglycine (l-Hag), into a protein through gene sequence design, followed by a free-radical hydrothiolation reaction with β-GlcSH. This process produced glycoconjugate protein **41** in almost quantitative yields, while retaining the protein stability and full functionality (Scheme 7). The reaction proceeded in aqueous solutions under irradiation at λ_max_ = 365 nm with Vazo 044 as the initiator. The method’s versatility was demonstrated by using both protected and unprotected thiosugars, as well as a range of proteins with diverse structures. In addition, the authors extended the reaction conditions to self-assembled multimeric Qβ-(Hag 16) protein, confirming the complete glycoconjugation of all 180 olefins with excellent chemoselectivity. This effective and complete site-selective glycoconjugation offers significant potential for preparing glycoconjugate vaccines, where high levels of loading are desirable.

**Scheme 7:**
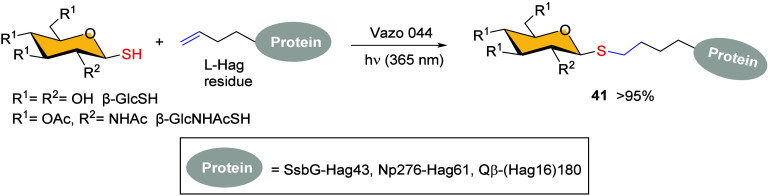
Protein S-Linked Glycoconjugation^a^

a Vazo 044 = 2,2′-azobis­[2-(2-imidazolin-2-yl)­propane] dihydrochloride.

Around the same time, Dondoni and co-workers reported a complementary strategy using the TEC to connect alkenyl *C*-glycoside to a peptide or protein containing SH-free cysteine groups. Initial studies demonstrated the accomplishment in TEC reaction between *C*-glycosides with an anomeric-allyl group and cysteine-containing peptides, such as the tripeptide glutathione (γ-Glu-Cys-Gly), using DPAP as radical initiator.^67^ The methodology was extended to couple *C*-galactoside **42** to thiol containing nonapeptide TALNCNDSL, and to globular bovine serum albumin protein (BSA), yielding glycoconjugates **43** and **44** respectively (Scheme 8). Although native BSA is known to contain only one cysteine residue (Cys-34), spectrometric analysis of glycosylated protein **44** revealed the presence of three galactoside rings. The two additional sugar rings resulted from the TEC reaction between the alkene-sugar ring and sulfhydryl groups generated by disulfide bridge cleavage at positions 75–91.

**Scheme 8:**
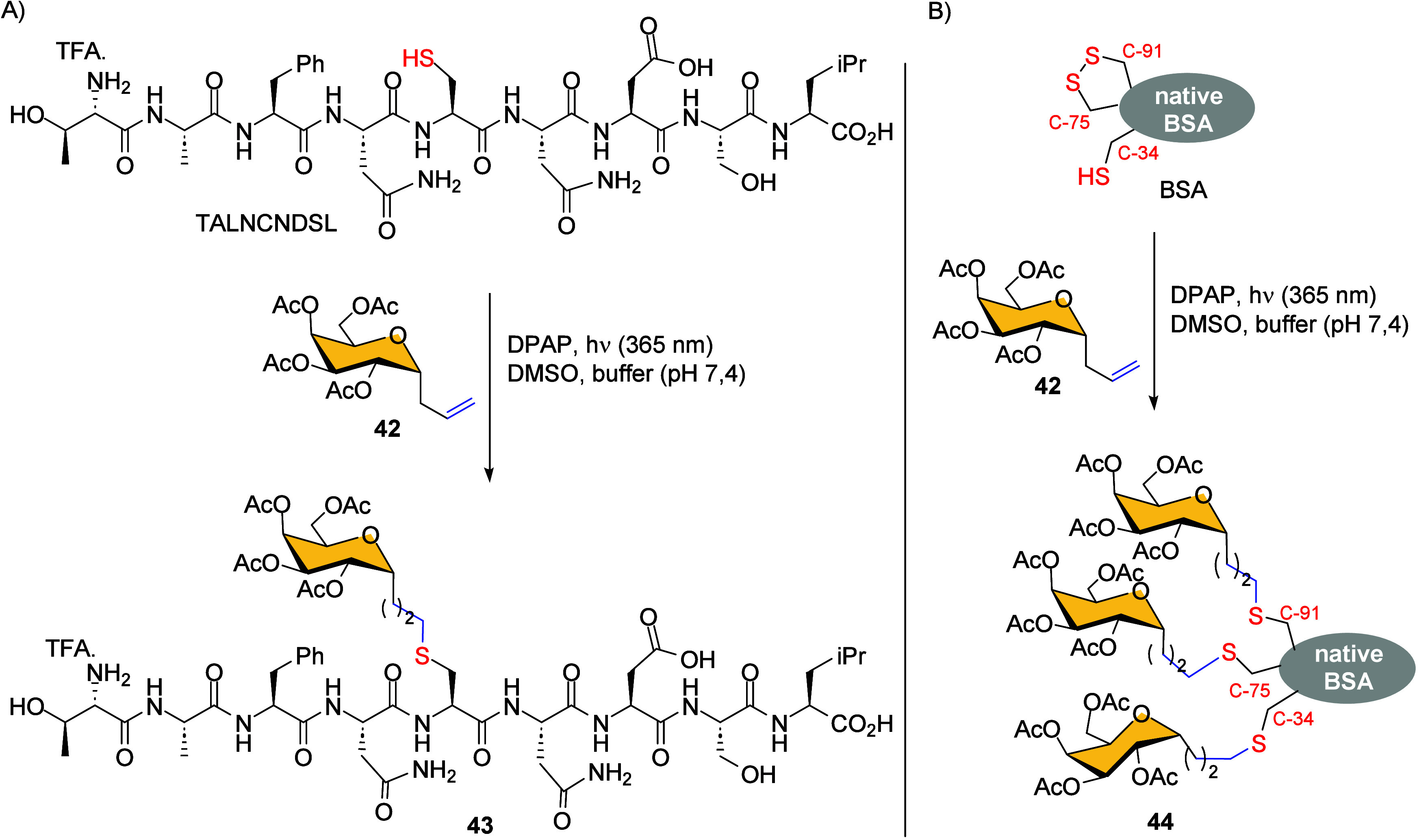
(A) Preparation of TALNCNDSL Glycoconjugate **43**; (B) Preparation of BSA Glycoconjugate **44**

A few years later, Deming and co-workers described the synthesis of novel conformation-switchable glycopolypeptides **46**, which undergo α-helix-to-coil transitions upon oxidation and exhibit exceptional water solubility in both conformational states.^68^ The preparation of glycopolypeptide **46** involved a polymerization process (Scheme 9), where monomer fragments were designed to contain a stable thioether linkage introduced by TEC using *C*-linked glycosides and l-cysteine derivatives. The TEC reaction efficiently produced glycosylated amino acids **45** in high yield, containing the desired residues for subsequent polymer formation.

**Scheme 9:**
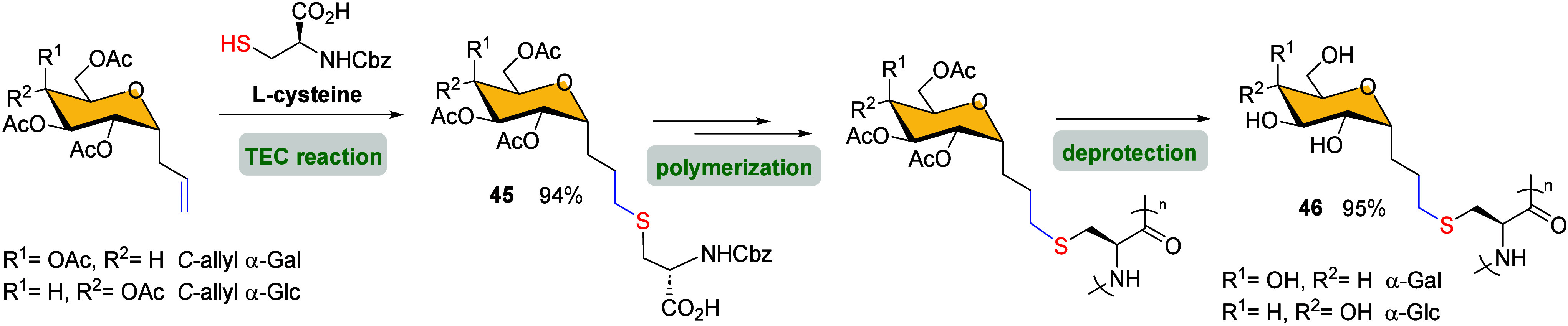
Synthesis of Glyco-C NCA Glycopolypeptides **46** Using TEC Reaction

Another interesting application of TEC was the chemical neoglycosylation of collagen patches, which are promising materials for incorporating therapeutic strategies. Cipolla et al. described an efficient procedure for preparing glycan-functionalized collagen **48** via a thiol–ene approach between alkene-derived monosaccharides and the thiol-derived collagen **47**.^69^ The reaction was carried out at room temperature in a MeOH:H_2_O (1:2) mixture under UV irradiation at 365 nm, with DPAP as the radical photoinitiator (Scheme 10).

**Scheme 10:**
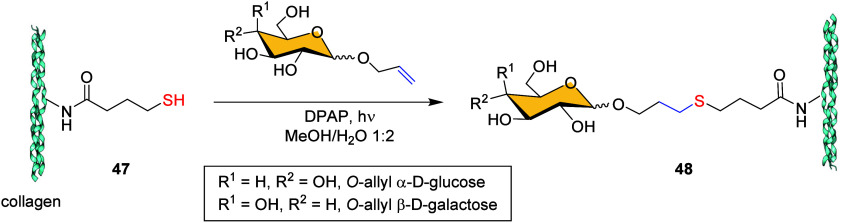
Thiol–Ene Reaction on Thiolated Collagen **47**

A significant advancement was made by using the photochemical TEC for glycoconjugate vaccine preparation, as previously proposed by Davis’ group.^66^ In comparison to saccharide conjugation, coupling glycopeptide antigens to proteins is more challenging due to the variety of functional groups involved. However, Kunz et al. reported the orthogonal end efficient preparation of a cancer vaccine by combining a synthetic tumor-associated glycopeptide antigen with a carrier protein through TEC.^70^ Thiol functionalized glycopeptide **49** and alkyne-terminating BSA protein **50** were subjected to photoinduced thioether conditions, generating a thioether spacer in synthetic vaccine **51**. The reaction proceeded in water at room temperature, and the Vazo 044 radical initiator significantly increased the reaction yield. An excess of thiol was necessary to provide the BSA conjugate vaccine **51**, which contained an average of eight molecules per BSA molecule (Scheme 11).

**Scheme 11:**
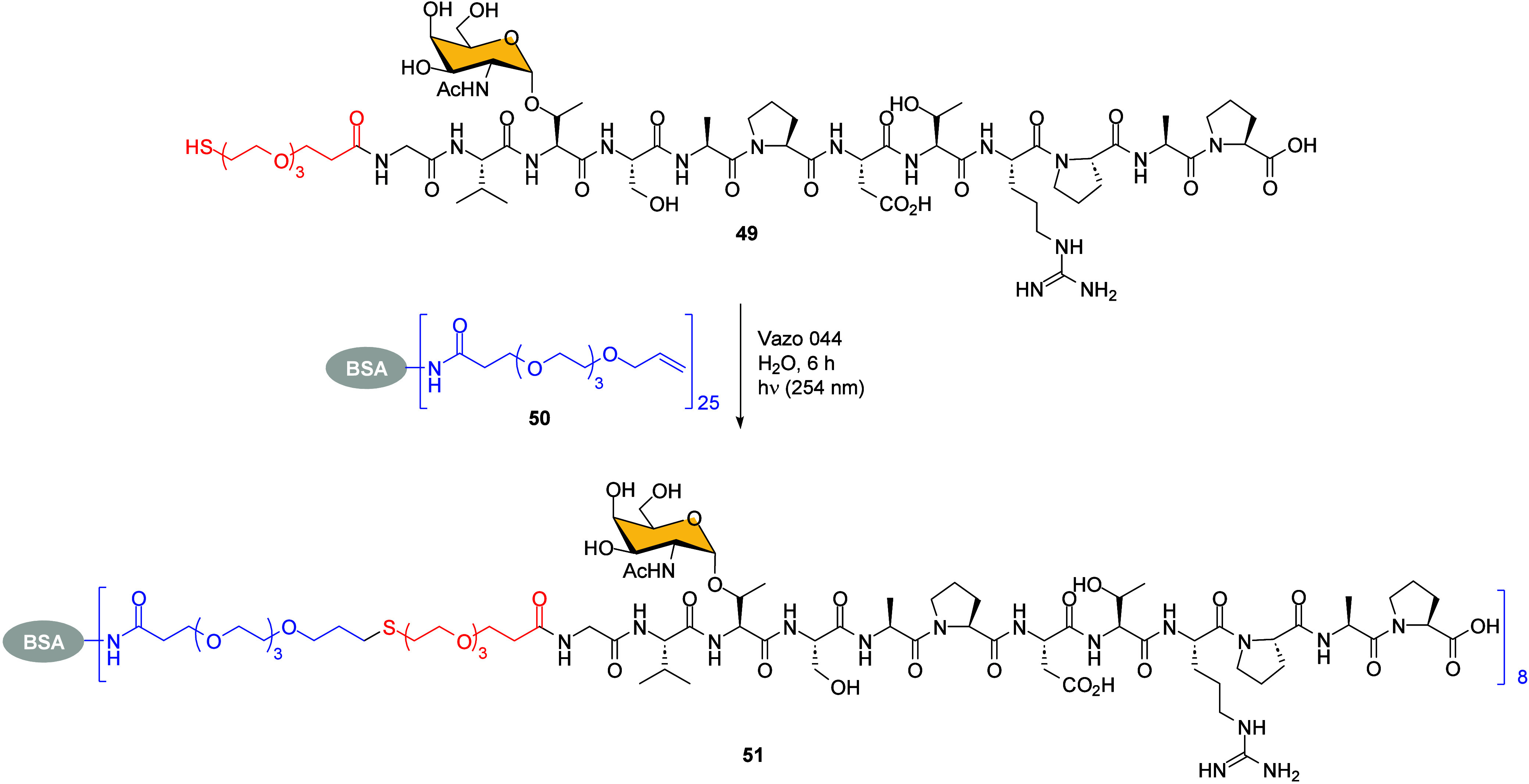
Photocatalyzed TEC-Ligation between a Thiolated Glycopeptide **49** and Allylated BSA Carrier **50**

The nonimmunogenic thioether linker generated by this methodology offers a promising opportunity to create vaccines that stimulate the immune system against cancerous cells.

#### 2.2.3 Synthesis of Glycodendrimers and Glycoclusters via TEC

Although protein–carbohydrate interactions are essential to many biological processes, individual interactions typically exhibit weak binding affinities. However, multiple interactions between multivalent ligands and receptors can significantly enhance the binding strength at the molecular scale. In this context, multivalent synthetic neoglycoconjugates with well-defined structures are potential inhibitors of natural oligosaccharide ligands and are useful tools for understanding such carbohydrate–protein interactions. The central core of a glycodendrimer serves as the focal point for the attachment of carbohydrate branches. This core can be composed of various materials, but it is typically a small organic molecule or a metal ion complex, which can influence the properties of glycodendrimers and functions.

Over the past decades, the photolytic thiol–ene reaction has emerged as a powerful method for synthesizing glycodendrimers.^71^ In 1997, Lindhorst and co-workers reported the first glycodendron synthesis using monosaccharides as multifunctional building blocks.^72^ The photoinduced TEC between perallylated α-d-glucopyranoside and cysteamine hydrochloride was carried out in MeOH, leading to a sugar-derived pentaamine core structure **52** in high yield. Further coupling with mannosyl isothiocyanate and subsequent *O*-acetyl deprotection afforded a thiourea-bridged pentaantennary glycocluster **53** (Scheme 12). Years later, the same group employed a similar strategy, consisting of sequential thiol–ene/glycosylation/deprotection reactions, to prepare a new family of d-glucose-centered mannosyl clusters with valencies as high as 15 branches. Their affinities toward the mannose-specific lectin concavalin A (Con A)^73^ and their potential as inhibitors of type 1 fimbriae-mediated adhesion of to mannose polysaccharide^74^ was evaluated. It was concluded that a higher valency correlated with higher biological activity.

**Scheme 12:**
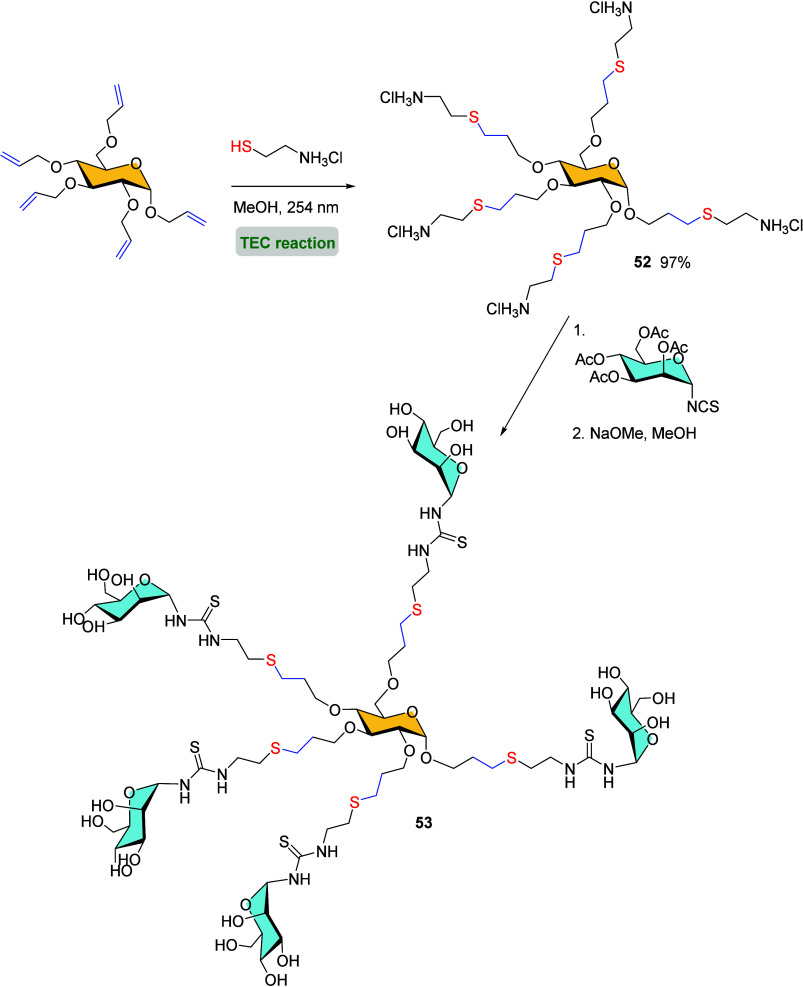
Synthesis of 5-Valent Glycocluster **53** *via* Sequential Thiol–Ene/Glycosylation Reactions

Polyhedral oligosilsesquioxanes (POSS) hybrid scaffolds, a class of nanoscale cage-like structures composed of silicon and oxygen atoms, often with organic functional groups attached to the surface, offer a highly versatile platform for functionalization.^75^ These unique structures make them ideal candidates for synthesizing multivalent glycoclusters. Many efforts have been made to efficiently functionalize commercially available octavinyl POSS **54** with different saccharides via radical-photoinduced TEC. In 2004, Lee and co-workers successfully conjugated the thiol group of glycosyl γ-thiobutyramides **55** to cluster **54** in the presence of catalytic amounts of AIBN (Scheme 13).^76^ The inhibitory effect of the prepared glycocluster **56** on human α1-acid glycoprotein by RCA120 (a β-galactose specific lectin) was evaluated, showing 200 times more activity than lactose, presumably due to the cluster effect of the binding of asialo-oligosaccharide. Dondoni and co-workers later studied the direct incorporation of 1-thio-β-d-glucose **58**, without a suitable spacer, to the octavinyl-POSS **54** under standard TEC conditions with DPAP. Unfortunately, a mixture of partially glycosylated POSS products was detected, likely due to steric congestion around the octasilsesquioxane scaffold during the sequential attachment of thioglycoside residues.^77^ To address this limitation, the authors introduced a suitable arm between the saccharide and the thiol group. Thiopropyl α-d-*C*-glucopyranose or *C*-mannopyranose provided the corresponding glycocluster in high yields under similar TEC conditions. Alternatively, the authors proposed a procedure to space out the cluster backbone and the alkene groups consisting on: (i) HS-PEG fragment attachment to octavinyl-POSS **54** via photoinduced coupling, (ii) treatment of the octahydroxy functionalized POSS **57** to generate the corresponding alkene functionalities, (iii) treatment with PEG-linked octaene silsesquioxane with 1-thio-β-d-glucose **58** by means of TEC to successfully afford octavalent *S*-linked glycocluster **59** (Scheme 13).

**Scheme 13:**
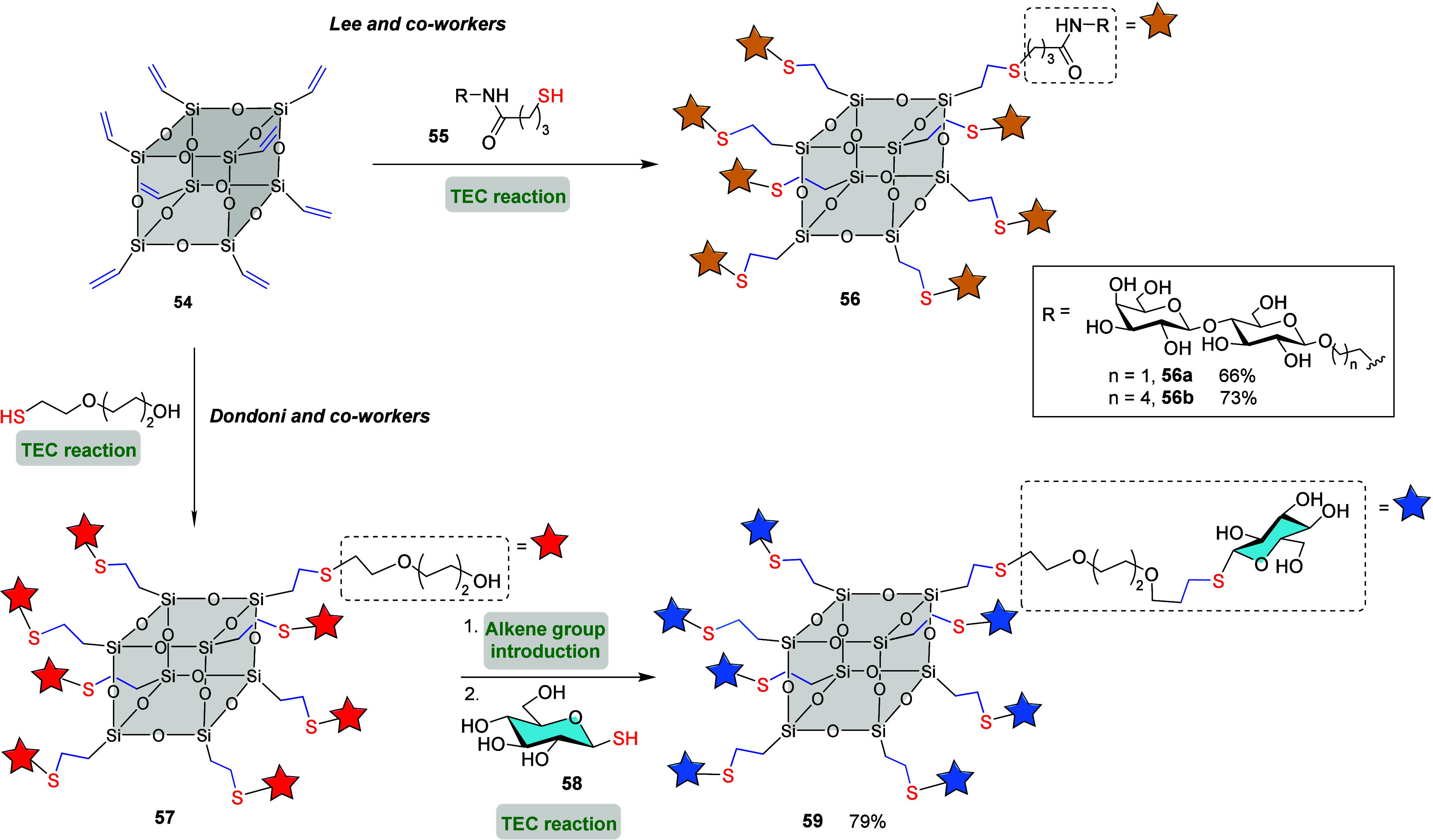
Synthesis of 8-Valent Glycocluster **56** and **59** Using POSS Platform and TEC Reaction

A different central scaffold based on aromatic cores was described by Dondoni and Hawker. They described the regioselective introduction of thio-sugar residues to the fourth generation alkene functional dendrimer [G4]-ene_48_, based on the tris-alkene core 2,4,6-triallyloxy-1,3,5-triazineas as aromatic dendritic platform.^78^ The TEC reaction conducted in DMF under irradiation at λ_max_ 365 nm in the presence of 2,2-dimethoxy-2-phenylacetophenone (DPAP), led to the quantitative conversion of all 48 alkene groups of the dendrimer within just 1 h. The resulting glycodendrimers **60**, grafting 48 units of glucose, mannose, lactose or sialic acid, were isolated in excellent yields without the need of protecting groups (Figure 1).

**Figure 1:**
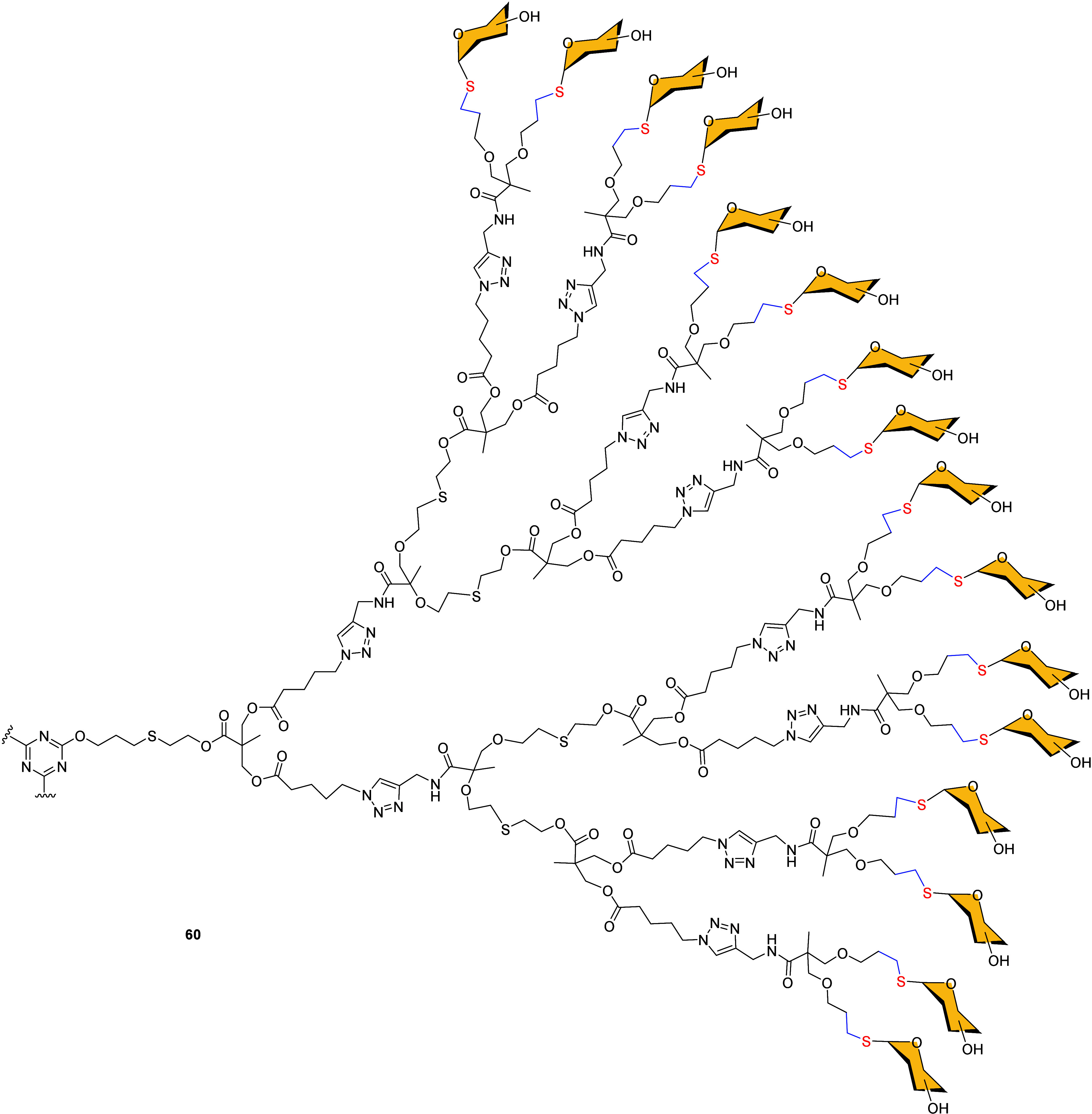
Glycodendrimers **60** are based on an aromatic 48-valency dendrimer [G4]-ene_48_ central core.

Later, Dondoni and co-workers applied a similar methodology to produce globular glycodendrimers holding sugar fragments via flexible thioglycosidic linkages by photoinduced coupling of 2-acetamido-2-deoxy-1-thio-β-d-glucose (GlcNAc-SH) to alkene functional polyester-based dendrimers. ELLA-based bioassays of the prepared glycodendrimers demonstrated excellent binding properties toward wheat germ agglutinin (WGA) compared to the monosaccharidic GlcNAc used as monovalent reference.^79^

An efficient protocol to prepare multivalent flexible trithiomannoside clusters **65**, which have been shown enhanced antimicrobial activity against Gram-negative bacteria, was reported by Chan-Park et al.^80^ The methodology involved a 3-step process: (i) coupling of tri-*O*-allyl compound **61** and 2,3,4,6-tetra-*O*-acetyl-1-thio-α-d-mannopyranose under TEC conditions using dimethoxyphenyl acetophenone (DMPA) as radical initiator, followed by Boc and acetyl deprotections to afford free amine trithiomannoside cluster **62**, (ii) amide coupling with the acid group in **63** using EDC·HCl and HOBt to obtain the tri-*O*-allyl terminated glycocluster **64**, (iii) finally, the terminal olefins were subjected to thiol–ene click reaction with 2,3,4,6-tetra-*O*-acetyl-1-thio-α-d-mannopyranose to give, after deacetylation, bis-trithiomannoside cluster **65** (Scheme 14).

**Scheme 14:**
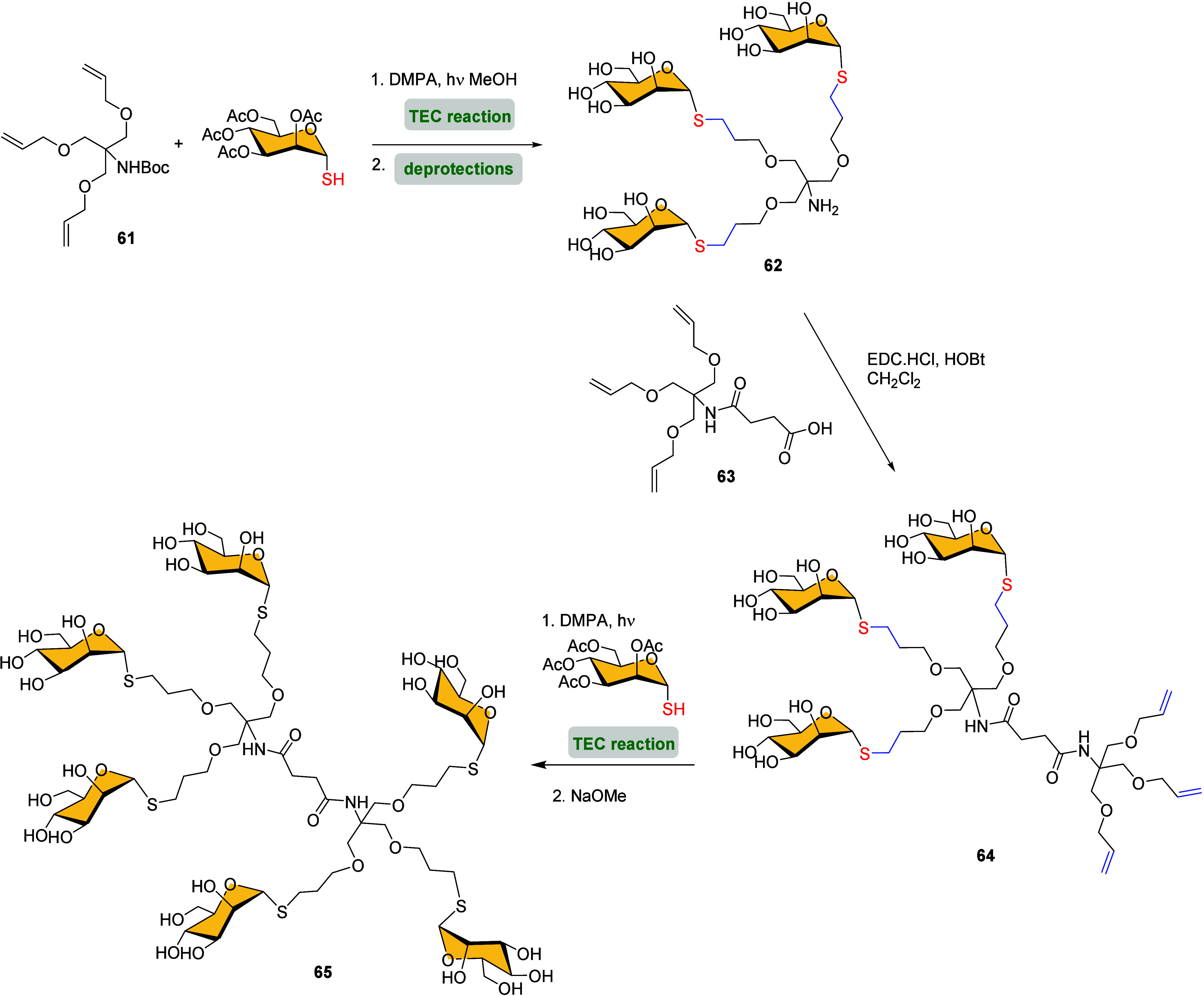
Synthesis of 6-Armed Glycodendrimer **65**

Cyclodextrins (CDs) are naturally occurring cyclic oligosaccharides that have been extensively studied due to their ability to form inclusion complexes with various guest molecules. Their unique structure, with a hydrophobic cavity and a hydrophilic exterior, allows them to encapsulate hydrophobic guest molecules, thereby improving their solubility, stability, and bioavailability. This property has been widely exploited in pharmaceutical drug formulations to enhance the delivery of poorly soluble drugs. However, the perfunctionalization of CDs is indeed a challenging task because of their complex structure. The TEC reaction offers advantageous properties for introducing new functionalities onto *O*-per-allylated cyclodextrins. In 1998, Roque and co-workers prepared polyanionic and polyzwitterionic cyclodextrins-based compounds as potential inhibitors of HIV transmission by radical addition of thiomalic acid or mercaptopropionic acid onto perallylated cyclodextrins (CDs) under UV irradiation with a catalytic amount of AIBN.^81^ Years later, Stoddart and co-workers used the TEC to decorate, either or both the primary and the secondary faces, of *O*-per-allylated cyclodextrins **66a** with glycosyl thiols to efficiently access carbohydrate free clusters **68a** in good yields after easy removal of protecting groups (Scheme 15).^82^

**Scheme 15:**
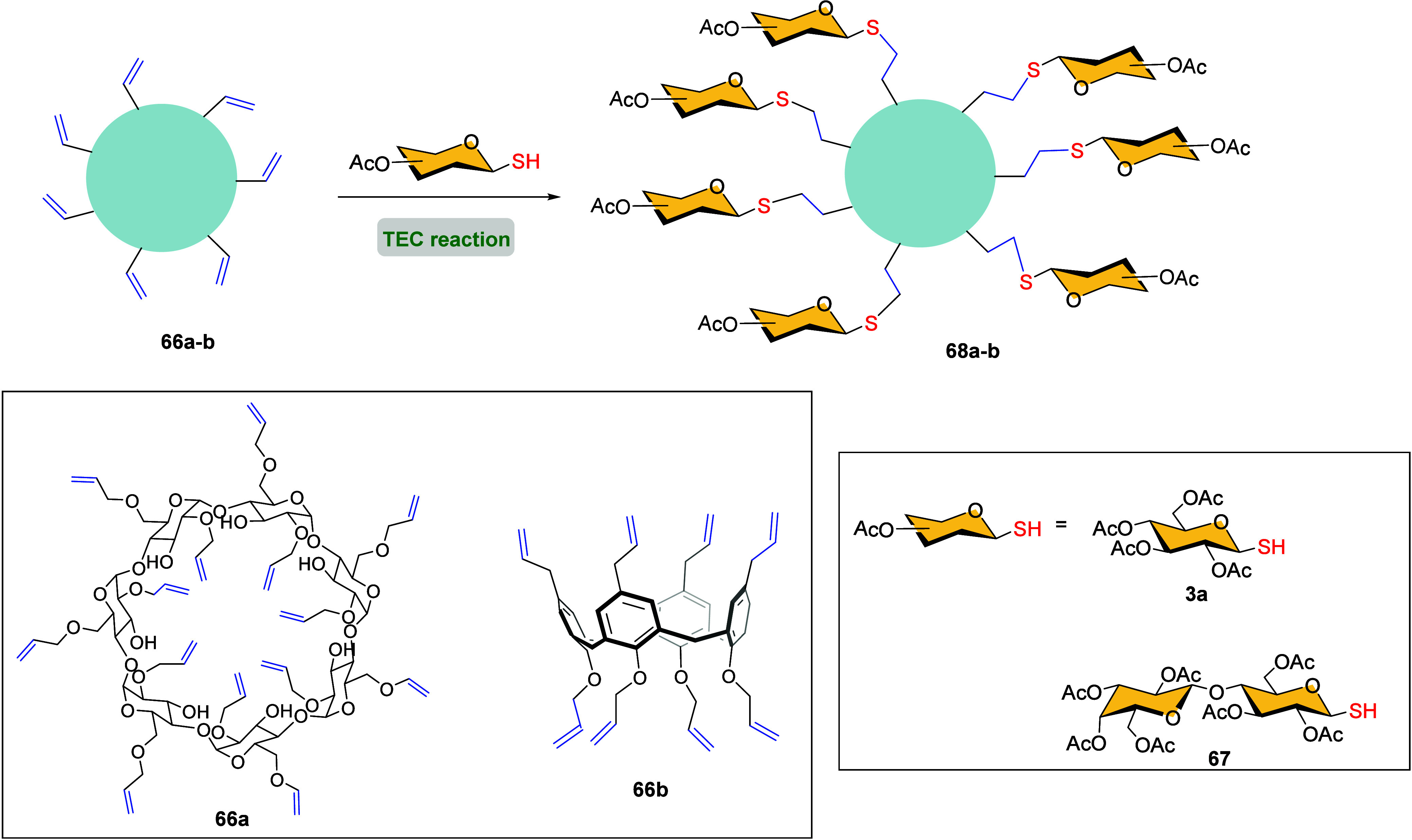
Preparation of Glycodendrimers **66a**–**b** by TEC Reaction

Calix­[4]­arenes have emerged as ideal central cores for preparing glycodendrimers due to their unique structural features. They are cyclic molecules composed of four aromatic rings connected by methylene bridges and provide a stable and well-defined platform. The first synthesis of calix[4]­arene-based *S*-glycoclusters via photoinduced multiple TEC was reported by Dondoni and co-workers in 2009.^83^ Reaction of alkene-functionalized calix[4]­arenes **66b** with sugar thiols by irradiation at λ_max_ = 365 nm in CH_2_Cl_2_ using DPAP as the sensitizer afforded the octavalent *S*-glycoside cluster **68b** in good yield (Scheme 15).

So far, the examples shown in this review refer to the synthesis of multivalent homogeneous glycoclusters via TEC reaction. However, the recognition process in nature often involves multiple types of sugar ligands. Therefore, the preparation of heteroglycodendrimers, where different types of sugar ligands are attached to the same dendritic scaffold, is crucial for understanding the roles of specific sugar moieties in biological recognition, and many efforts have recently been made in this context.^84^ García Fernándeźs group prepared heptavalent heteroglycoclusters **73** by subsequent photoinduced radical addition (Scheme 16).^85^ Initially, two units of per-*O*-acetylated 1-thiosugars (yellow colored in scheme) were added using TEC reaction to the tri-*O*-allylated pentaerythritol derivative **69** by irradiation at 250 nm in MeOH. The relative quantities of the sugar moiety must be carefully regulated to achieve specific outcomes, leading to either mono- or di- derivatives. Then, divalent derivatives **70** were subjected to the same TEC conditions using a different thiosugar (blue colored in scheme), resulting in the effective formation of bifunctional ligands **71**. The remaining primary hydroxy group was further converted into an isothiocyanate group, which was coupled to the face-selective functionalized per­(C-6)-heptacysteaminyl-β-CD derivative through thiourea bridges. Subsequent deacetylation led to the heptavalent heteroglycoclusters **73** containing both α-Man β-Glc and α-Man β-Lac saccharides. Biological assays showed significantly higher binding affinity of heterocluster **73** to Con A compared to analogous homogeneous conjugates with the same number of mannose units. This higher binding affinity suggests that the presence of multiple sugar types in the heteroglycoclusters leads to more effective clustering effects, resulting in enhanced interactions with Con A on a mannose molar basis (up to 8-fold increase in affinity).

**Scheme 16:**
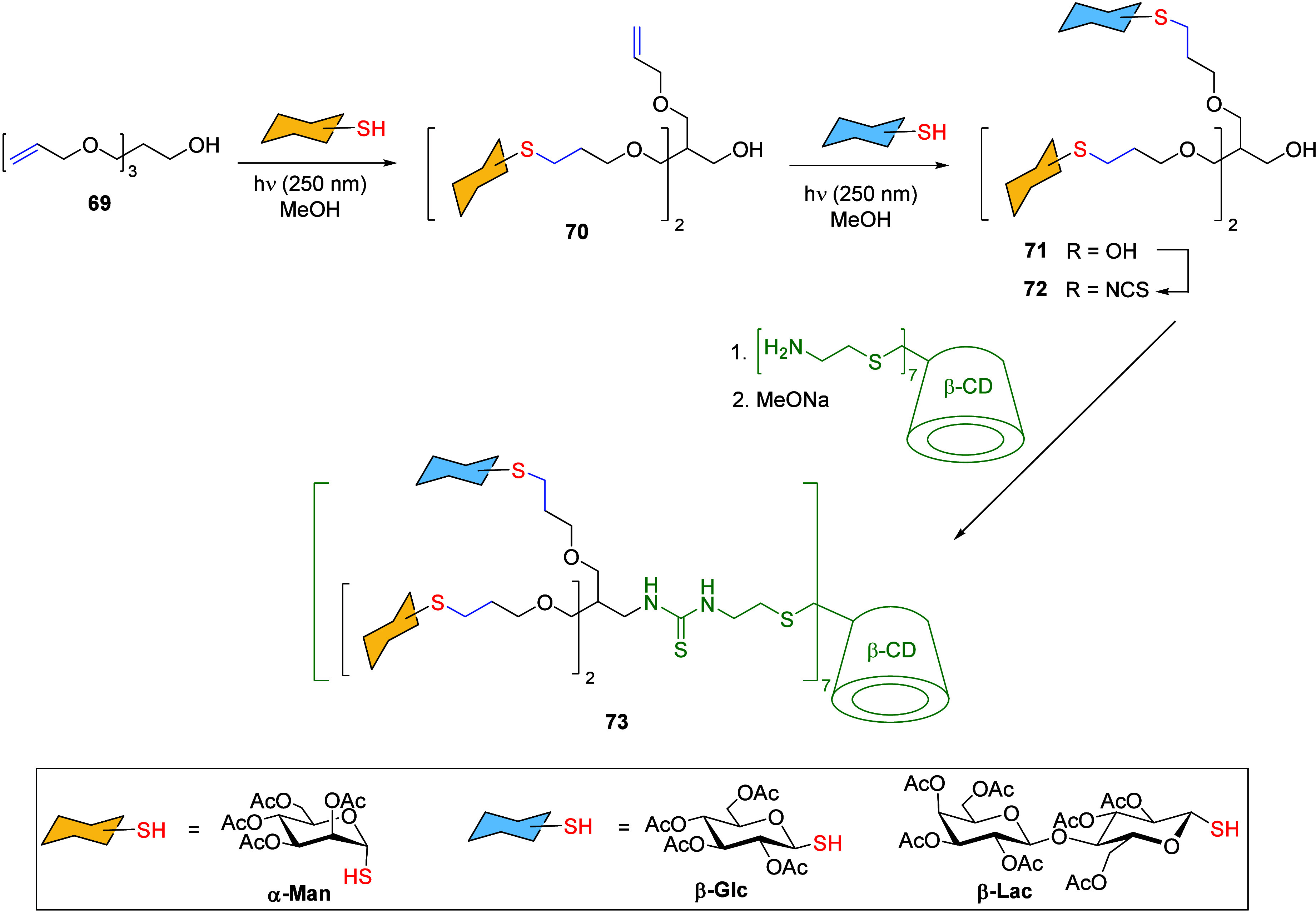
Preparation of Heteroglycoclusters **73** *via* TEC

Later the authors extended their work on heteroclusters,^86^ and other research groups have also contributions to the development of efficient methodologies for preparing heteroclusters.^83,87^

We cannot overlook the thiol–yne coupling (TYC) reaction, which has emerged as an important and widely utilized synthetic method. The reaction involves the coupling of one or two thiols across a C–C triple bond via free-radical chain mechanism (similar to TEC). Due to its compatibility, efficiency, and mild reaction conditions, TYC continues to be an active area of research with potential applications in bioconjugation chemistry.^88^

In summary, the thiol–ene coupling reaction has gained significant attention due to its efficiency, selectivity, high yield, and fast reaction kinetics, making it a powerful tool for developing new glycoconjugates and biomaterials. Bioconjugation using this methodology has enabled the introduction of glycans to various sensitive biomolecules in the presence of a wide range of functional groups found in nature, confirming the reaction’s biocompatibility. Moreover, TEC is a biologically friendly coupling reaction that requires benign radical initiators, avoiding the use of toxic metal catalysts or reagents. However, the potential use of this methodology *in vivo* experiments is limited by the undesired reaction with thiol functions in cells, and the low tolerance of cells to high energy UV irradiation.^89^ Therefore, the bioorthogonality of the thiol–ene coupling is limited, and alternative strategies should be considered for bioconjugation in living organism.

## 3 Strain-Promoted Azide-Alkyne [3 + 2] Cycloaddition (SPAAC)

### 3.1 Introduction

In early 2002, Meldal and colleagues developed a catalytic version of the classical Huisgen 1,3-dipolar cycloaddition,^90,91^ which requires copper­(I) salts and terminal alkynes, producing stable 1,4-disubstituted 1,2,3-triazoles in a rapid and regioselective manner through a stepwise mechanism (Scheme 17A).^92^ This reaction, known as CuAAC, perfectly aligns with the criteria established by Sharpless, Kolb, and Finn in 2001 for a click-type reaction.^15^ Despite its widespread use and effectiveness demonstrated across various research fields, including chemical biology and drug design, the use of CuAAC for biological and medicinal applications is considerably restricted by the cytotoxicity of copper. In this sense, the development of a metal-free [3 + 2] cycloaddition that retains the advantageous properties of CuAAC while eliminating the necessity for metal catalysts is particularly essential. Bertozzi et al.,^93–96^ and later other authors,^38,97^ developed a version of the [3 + 2] cycloaddition between organic azides and strained cyclooctynes as a bioorthogonal alternative to CuAAC (Scheme 17B). This reaction, named SPAAC, can be performed efficiently under physiological conditions and in living organisms, tolerating the presence of various functional groups commonly found in biological systems. Therefore, this approach meets the criteria for bioorthogonality,^36^ addressing the limitations that other click reactions, such as the TEC discussed earlier in this review. SPAAC offers high selectivity, versatility, biocompatibility, and fast reaction kinetics.

**Scheme 17:**
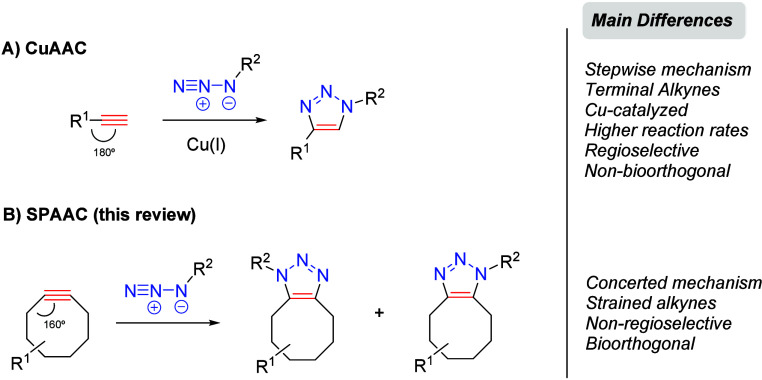
Comparison of the CuAAC and the SPAAC Reactions

Despite SPAAC embodying the core principles of a click transformation, this approach has some drawbacks compared to Cu-catalyzed [3 + 2] cycloaddition: (i) the lack of regioselectivity, resulting in two possible regioisomers in similar ratios; (ii) the synthetic complexity of cyclooctynes, which shows a clear relationship between reactivity and synthetic difficulty; and (iii) the need to functionalize cyclooctynes for conjugation with other molecules or probes.

Cyclooctynes present a fine equilibrium between stability and reactivity, making them well-suited for SPAAC reactions.^98,37,99^ A plethora of structurally diverse cyclooctyne scaffolds have been prepared and studied for cycloaddition with azides; however, discussing all the cyclooctyne variants that have been applied in SPAAC reactions goes beyond the scope of this review.^37,100^

The desirable features mentioned above have led to increased interest in applying SPAAC in carbohydrate chemistry for the bioorthogonal functionalization of biomolecules with probes in biological systems and living organisms as well as for the efficient preparation of glycoconjugates and glycomimetics under mild conditions.

### 3.2 Applications

#### 3.2.1 SPAAC Applications for in Vivo Cell Imaging

Bioorthogonal chemical reactions that enable rapid and selective biomolecule labeling in living organisms have become powerful tools for probing biological processes *in vivo*. Apart from Staudinger ligation,^101–104^ there are very few examples of reactions that meet the bioorthogonality requirement,^95^ and among these, the SPAAC has recently emerged as the best candidate for the covalent direct conjugation of biomolecules with probes in biological systems and living organisms. In 2006, Bertozzi et al. first developed the bioorthogonal chemical reporter strategy to visualize cell-surface glycans, which cannot be easily visualized using standard molecular imaging tools, in a two-step procedure: (i) selected azide-functionalized monosaccharides are metabolized by cells and subsequently incorporated into cell-surface glycans, a process known as metabolic oligosaccharide engineering; and (ii) the azide-containing glycans are then reacted with an imaging probe-conjugated cyclooctyne through Cu-free click chemistry, enabling visualization of cell-surface azidosugars (Scheme 18).^95^ A series of carbohydrates have been used as starting material to prepare azido-containing carbohydrates (ManNAz, SiaNAz, GalNAz, XylAz and FucAz) as metabolic precursors for cell labeling via the SPAAC reaction to image glycans in living systems.

**Scheme 18:**
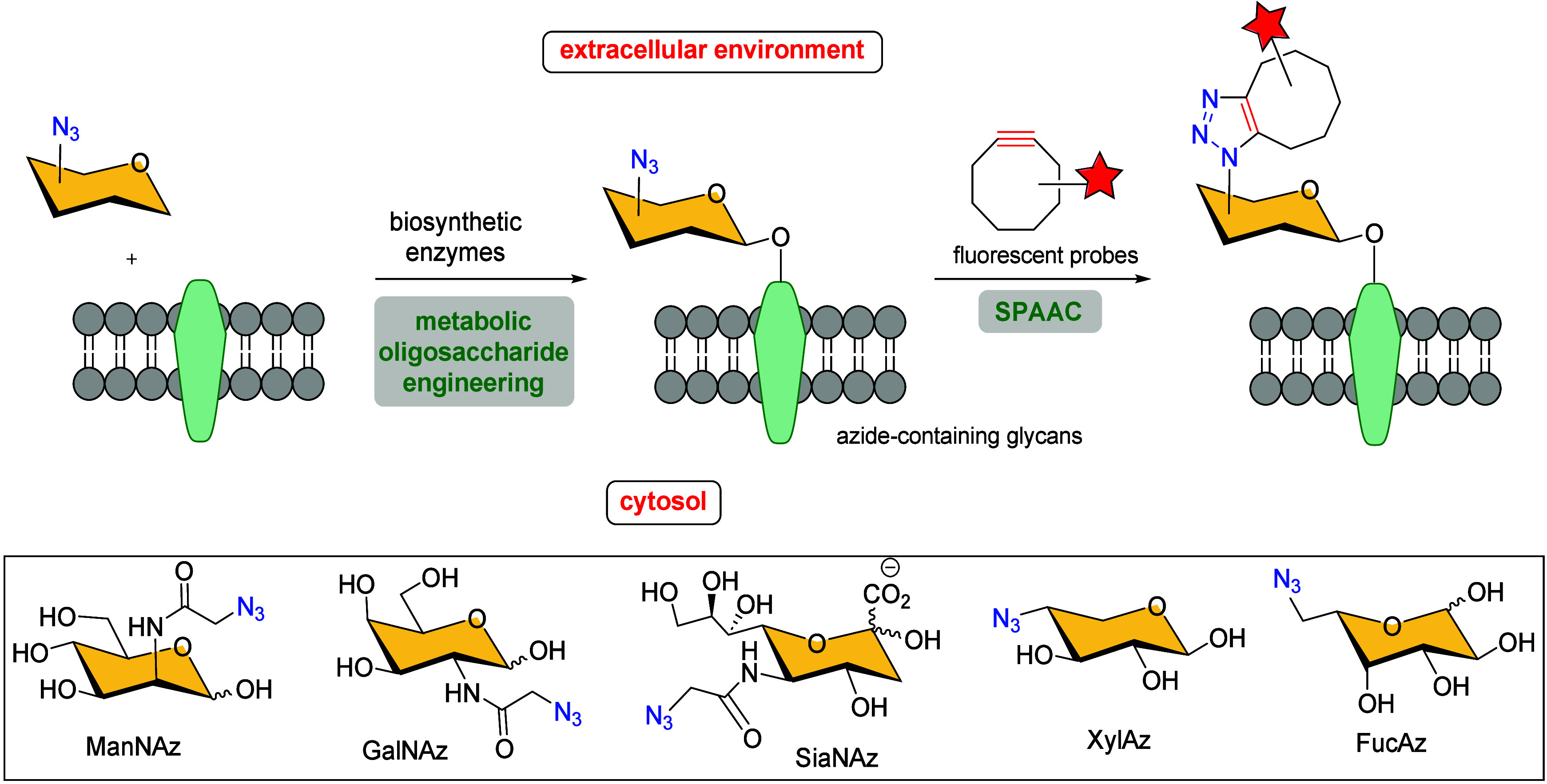
Imaging Cell-Surface Azidosugars with Cyclooctyne Probes *via* SPAAC Reaction

In this context, Bertozzi’s group applied the bioorthogonal [3 + 2] cycloaddition to rapid and selective label live cell surfaces glycans containing sialic acids, a family of negatively charged monosaccharides frequently expressed as external terminal residues on cell-surface.^93^ The metabolic incorporation of *N*-azidoacetyl sialic acid (SiaNAz) into cell-surface glycoproteins was achieved by treatment with Ac_4_ManNAz, and the resulting azide-labeled cells were then reacted with a series of fluorescent probe-conjugated cyclooctynes DIFO-R *via* a Cu-free click reaction (Scheme 19).

**Scheme 19:**
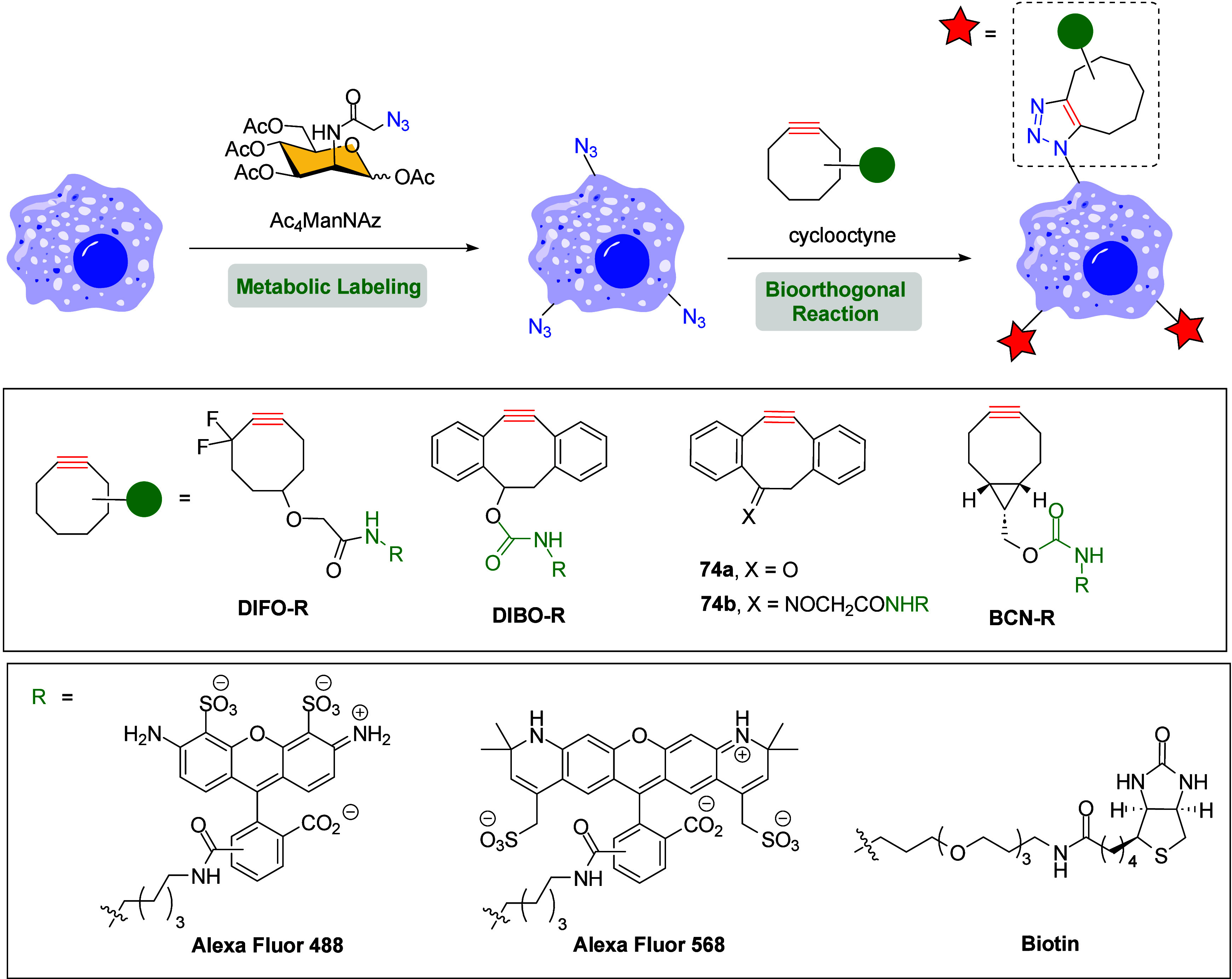
Metabolic Labeling and Visualization of Cell-Surface Glycans Using Ac_4_ManNAz and Different Functionalized Cyclooctynes with Fluorescent Probes

The DIFO (difluorinated cyclooctyne) reagent, containing two electron-withdrawing fluorine atoms, dramatically enhanced the selectivity and rate of the cycloaddition reaction compared to those of other cycloalkynes and other bioorthogonal ligations. Additionally, control experiments showed similar kinetic rates for the SPAAC reaction compared to the CuAAC version, performing effectively on live Jurkat cells. More interestingly, this nontoxic SPAAC approach enabled dynamic multicolor imaging of biochemical processes, and the internalization and trafficking of a population of labeled sialoglycoconjugates in live Chinese hamster ovary (CHO) cells were effectively monitored.

Around the same time, Boons et al. described a similar study to Bertozzi’s using 4-dibenzocyclooctynol (DIBO) as the crucial agent to which fluorescent probes were attached (Scheme 19).^38^ The aromatic moieties in DIBO provide additional ring-strain, increasing its reactivity with azides *via* SPAAC reaction compared to nonaromatic cyclooctyne analogues. Cells were cultured in the presence of Ac_4_ManNAz, resulting in the metabolic incorporation of SiaNAz into their cell-surface glycoproteins. This was followed by the SPAAC reaction with biotin-based DIBO and subsequent treatment with avidin-Alexa Fluor 488. This two-step cell-surface labeling approach showed higher kinetic rates and fluorescence intensities than those observed for the Staudinger ligation in a comparative study enabling the monitorization in real time of the trafficking of glycoproteins in living CHO cells. Years later, the authors described two modified DIBO structures incorporating a ketone (**74a**) or oxime group (**74b**) and evidenced that the biotin-modified **74b** and DIBO were useful probes to determine relative quantities of cell surface sialylation of wild-type and mutant cells (Scheme 19).^105^ Successfully, the SPAAC reaction of biotinylated DIBO reagents with metabolically labeled azido-bearing monosaccharide not only allowed the determination of relative quantities of sialic acid of living cells but also, in combination with lectin staining, revealed defects in the glycan structures of glycoproteins (Lec CHO cells).

Van Delft and co-workers applied the bioorthogonal SPAAC labeling methodology to study the bioavailability and tolerability of imaging surface glycans on living human melanoma MV3 cells,^97^ a class of highly invasive and metastatic cells in which the abundant production of surface glycans has been reported to play a role in invasion processes. MV3 melanoma cells were incubated with Ac_4_ManNAz, labeled with BCN-biotin conjugate through a nontoxic SPAAC protocol, and stained with streptavidin-Alexa Fluor 488 to visualize the redistribution of glycans during invasive cell migration. It is noteworthy that the synthesis of bicyclo[6.1.0]­nonyne (BCN) was straightforward and high yielding and exhibited relative stability. Moreover, the free metal cycloaddition reaction between BCN-biotin and glycan azides proceeded in excellent kinetic rates and led to a single regioisomer, due to the symmetric structure of BCN (Scheme 19).

More recently Cheng et al. designed two novel derivatives of the above-mentioned Ac_4_ManNAz that incorporate fatty acid esters (C_6_ and C_12_) on the anomeric hydroxyl group. These derivatives were encapsulated in a liposome delivery system to improve the chemical stability and the cell labeling efficiency (Scheme 20).^106^ Both Ac_4_ManNAz analogous **75a** and **75b** showed enhanced chemical stabilities and strong fluorescence intensity after forming the triazole rings using the azadibenzocyclooctyne DBCO-Cy5 fluorescent probe *via* the SPAAC reaction. However, the metabolic labeling efficiency on MDA-MB-231 cells was retained compared with Ac_4_ManNAz and appeared to be dependent on the length of the ester on the anomeric carbon, with compound **75b** performing better.

**Scheme 20:**
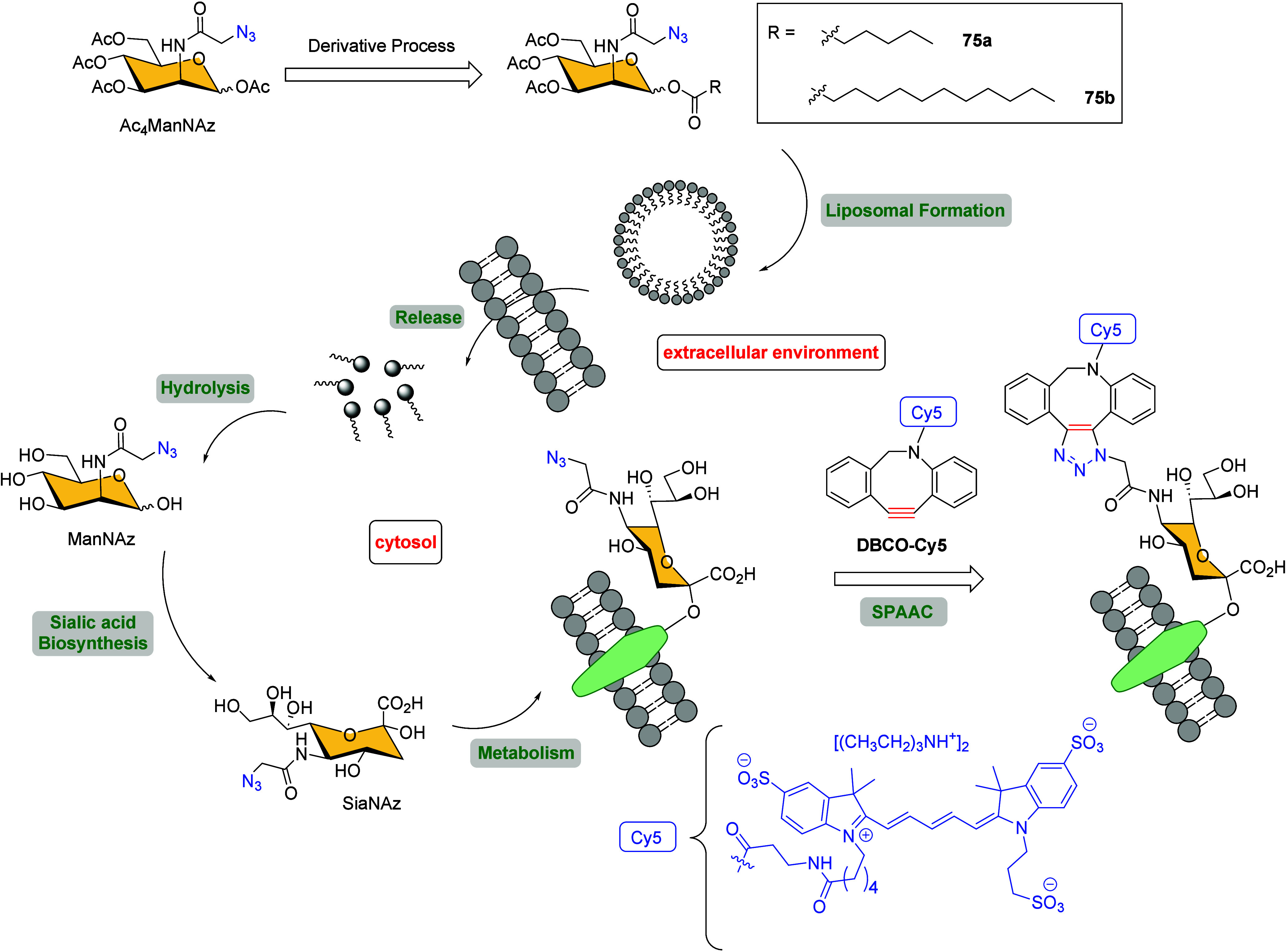
Metabolic Cell Labeling and Imaging Using Liposomal Azido Mannosamine Lipids

#### 3.2.2 SPAAC Applications for Living Organism Imaging

Apart from living cells, other more complex organisms have been used to study the efficiency of bioorthogonal reactions. Bertozzi et al. explored the SPAAC protocol for noninvasive imaging of glycans in live zebrafish during embryogenesis using fluorophore-DIFO conjugates.^107^ Ac_4_GalNAz was used as a metabolic label precursor to selectively incorporate azide groups into cell-surface glycans of zebrafish embryos. The metabolically labeled mucin-type O-glycans were subsequently reacted with fluorescent probe-conjugated DIFO *via* Cu-free click chemistry, allowing for the visualization of glycans *in vivo* at subcellular resolution (Scheme 21).^107^ This approach showed spatiotemporal changes in glycan distribution throughout zebrafish embryogenesis. Further investigations enabled the visualization of glycans dynamics in the enveloping layer during the early stages of embryogenesis, as early as 7 h post fertilization, using this methodology.^108^

**Scheme 21:**
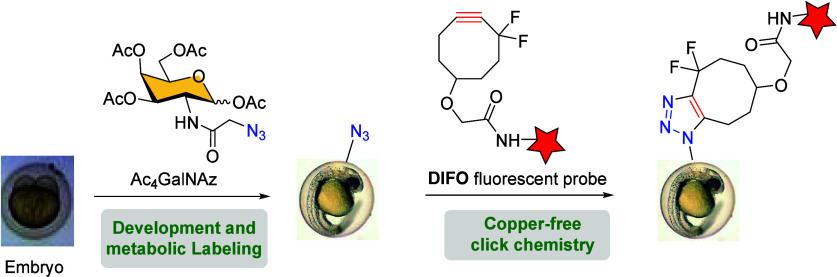
Noninvasive Imaging Strategy of Glycans in Live Developing Zebrafish

Prompted by the involvement of fucosylation in many developmental processes in living organisms, Bertozzi’s group explored the monitoring of fucosylated glycans in developing zebrafish using a strategy previously mentioned.^109^ The administration of an unnatural azide-functionalized fucose derivative, such as FucAz or FucAz-1-P, as metabolic substrates to label glycoproteins was inefficient, despite the presence of fucose salvage pathway enzymes during zebrafish embryogenesis (Scheme 22). This limitation was overcome by using the nucleotide sugar GDP-FucAz, a substrate for fucosyltransferase enzymes (FucTS), which allowed the incorporation of FucAz into glycoproteins in the Golgi lumen. These modified glycoproteins were then exported to the cell surface. To visualize FucAz incorporated into cell-surface glycans, the azide was reacted with the DIFO-488 fluorescent probe, allowing for the successful imaging of fucosylated glycans, which are difficult to monitor *in vivo* in the enveloping layer of zebrafish embryos during early development.

**Scheme 22:**
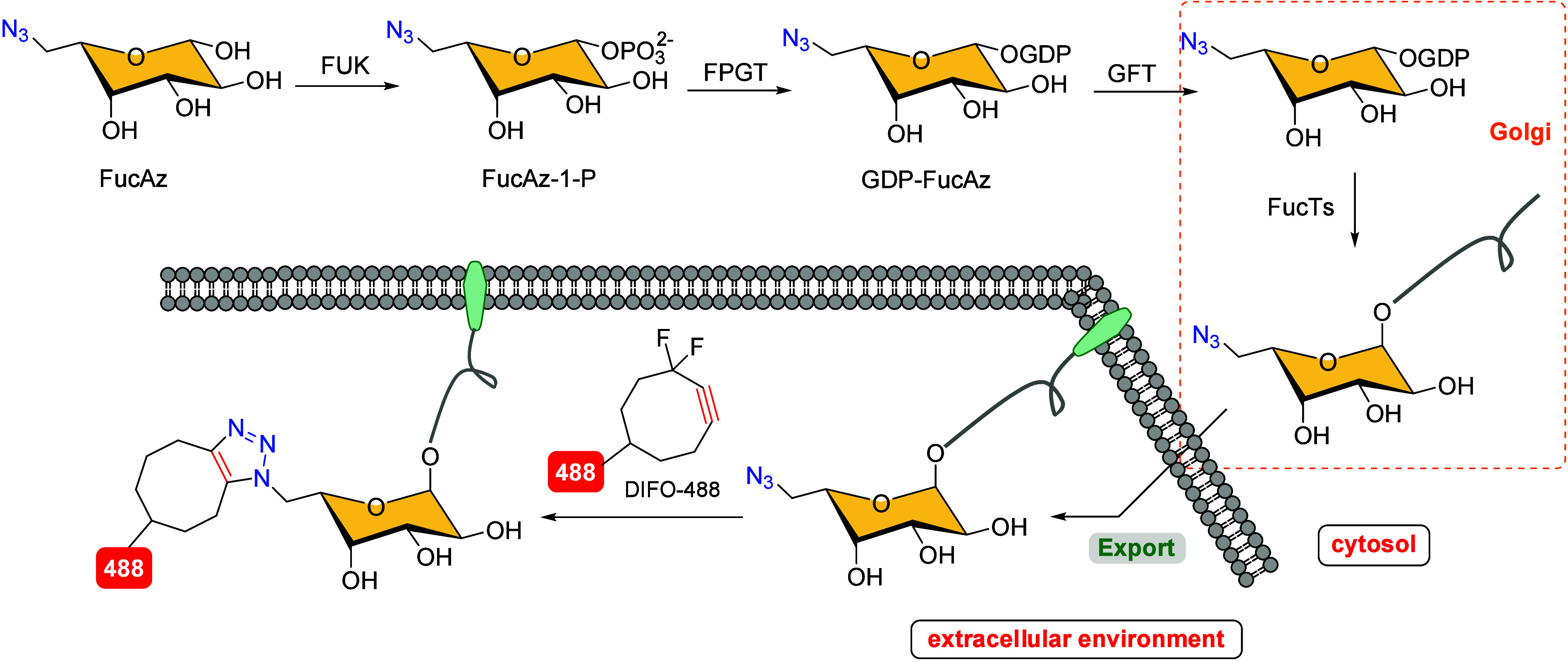
Metabolic Labeling of Fucosylated Glycans^a^

a FUK = fucose kinase, FucAz-1-P = FucAz-1-phosphate, FPGT = fucose-1-phosphate guanyltransferase, GDP = guanosine diphosphate.

Later, the same group again used a developing zebrafish as a model system to design a chain-terminator of glycosaminoglycan (GAG) biosynthesis, which represents the carbohydrate fraction of proteoglycans with crucial biological functions in animals (Scheme 23A).^110^ Taking into account that heparan sulfate (HS) and chondroitin sulfate (CS) glycosaminoglycans contain a conserved xylose residue (in blue in the scheme) that initiates the polysaccharide chain from the protein backbone, the authors metabolically replaced this monosaccharide with an unnatural azide-bearing xylose (4-XylAz) residue as a chemical chain-truncating analogue to probe GAG functions during zebrafish embryogenesis. Zebrafish embryos were exposed to UDP-4-azido-4-deoxyxylose (UDP-4-XylAz) to facilitate its incorporation into sites of GAG glycosylation, and the resulting embryos were allowed to develop (Scheme 23B).

**Scheme 23:**
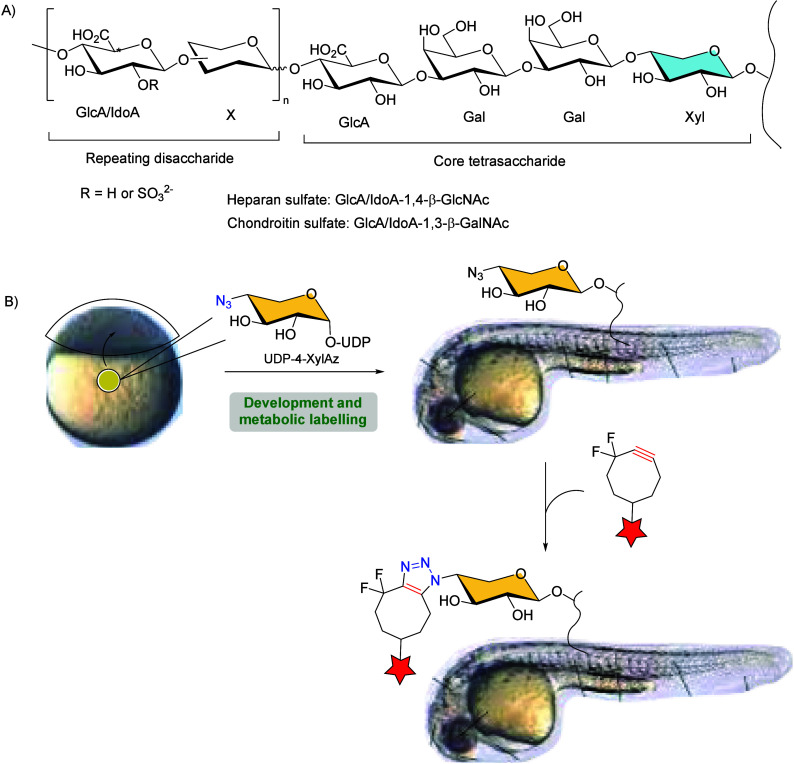
(A) Heparan Sulfate and Chondroitin Sulfate Structures; (B) Visualization of the GAG Inhibition Site Using a Fluorescent Probe-Conjugated Cyclooctyne^a^

a UDP= uridine diphosphate.

The azide labeled zebrafish embryos were then reacted with difluorocyclooctyne DIFO-AlexaFluor 488 to enable the rapid, efficient, and selective visualization of the GAG inhibition site *in vivo* through the SPAAC reaction. The copper-free click chemistry approach supplements genetic strategies for studying GAG function in living systems.

To extend the scope of the biorthogonal SPAAC approach for imaging metabolically labeled glycoconjugates in living systems, Bertozzi’s group first applied this strategy in the context of human tissue cultured *ex vivo* in 2019.^111^ High levels of sialylated glycans have been found in many types of cancer cells; however, the specific glycoproteins involved in cell-surface sialylation are not well characterized in human disease tissue. Therefore, the authors monitored glycoproteins located at the surface of cancerous prostate tissues using the mentioned methodology. Ac_4_ManNAz was again used as a biosynthetic precursor of azidosialic acid to be metabolized and incorporated into cell surface as well as secreted sialoglycoproteins of both normal and cancerous prostate tissues. Biotinylation followed by mass spectrometry techniques allowed the identification of the cell surface and secreted glycoproteins. It was found that cancerous prostatic tissues contained exceptionally high levels of glycoproteins compared to normal tissues. These studies established the utility of the SPAAC reaction as an essential part of metabolic labeling strategies to address questions of biomedical relevance.

Similarly, the SPAAC approach has been applied to other more complex living systems such as mice.^112^ Ac_4_ManNAz was metabolically incorporated in live mice to label their cell-surface sialic acids with azides, followed by the reaction with various cyclooctyne-FLAG peptide probes (Scheme 24). After the injection of cyclooctynes, labeled glycoconjugates were observed in various tissues, including the intestines, heart and liver, with no apparent toxicity. DIFO was identified as the cyclooctyne with the best intrinsic reactivity, although its bioavailability was a concern due to significant observed serum albumin binding. These studies establish SPAAC as an alternative bioorthogonal reaction to the Staudinger ligation that can be applied in live mice.

**Scheme 24:**
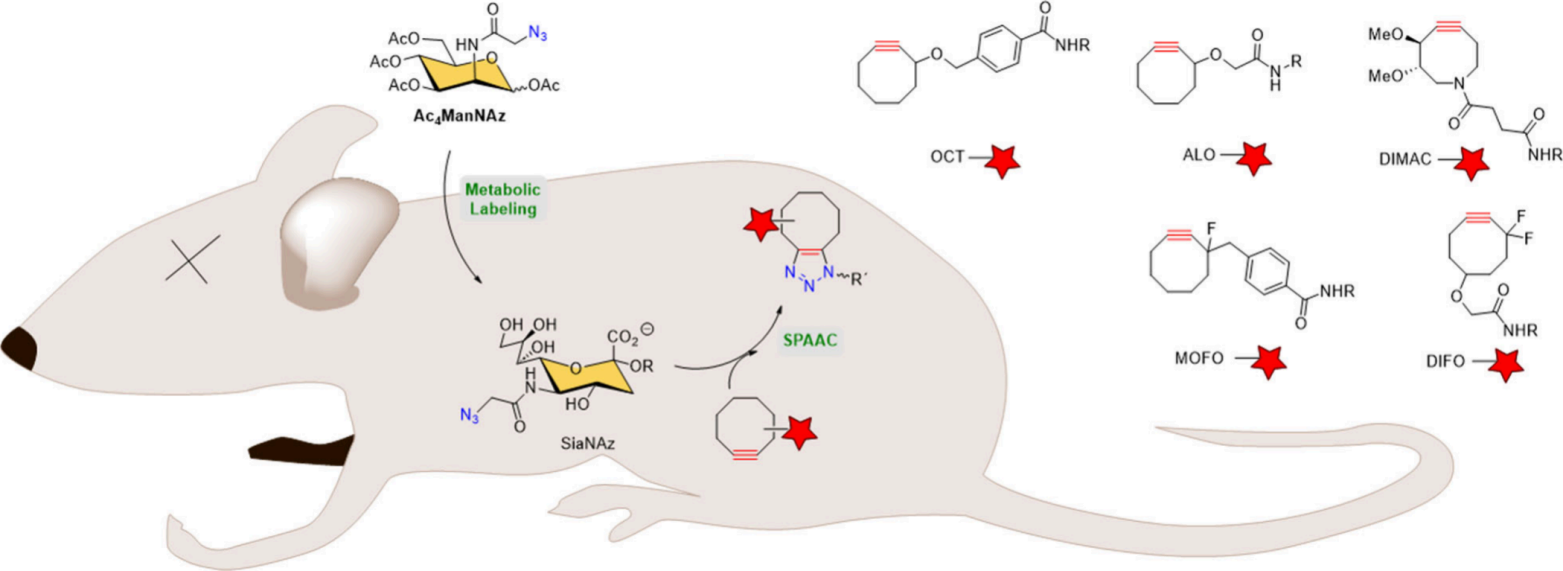
SPAAC in Mice^a^

a Mice were treated with Ac_4_ManNAz for metabolic labeling of glycans with SiaNAz. A series of cyclooctyne-FLAG conjugates were assayed for *in vivo* covalent labeling of azido glycans. R = FLAG peptide.

Although the mammalian brain is an organ rich in sialoglycans that play key roles in brain development, cognition, and disease progression, *in vivo* visualization of sialoglycan biosynthesis is a challenge due to the blood–brain barrier (BBB). A significant advance in this area was made by Chen et al., who designed a liposome-assisted bioorthogonal reporter (LABOR) strategy via SPAAC for metabolic labeling and visualization of brain sialoglycans in living mice.^113^ The authors demonstrated that liposomes encapsulating 9-azido sialic acid (9AzSia) can cross the BBB, delivering the azidosugar into the brain for the metabolic labeling of sialoglycoconjugates (Scheme 25). The resulting 9AzSia-labeled glycoconjugates were subsequent reacted with azadibenzocyclooctyne-Cy5 conjugate (DBCO-Cy5) as a fluorescent probe through Cu-free click chemistry, leading to the fluorescence imaging of brain sialoglycans in living mice and brain sections. The LABOR strategy enabled *in vivo* visualization of brain sialoglycan turnover, which is spatially regulated in distinct brain regions.

**Scheme 25:**
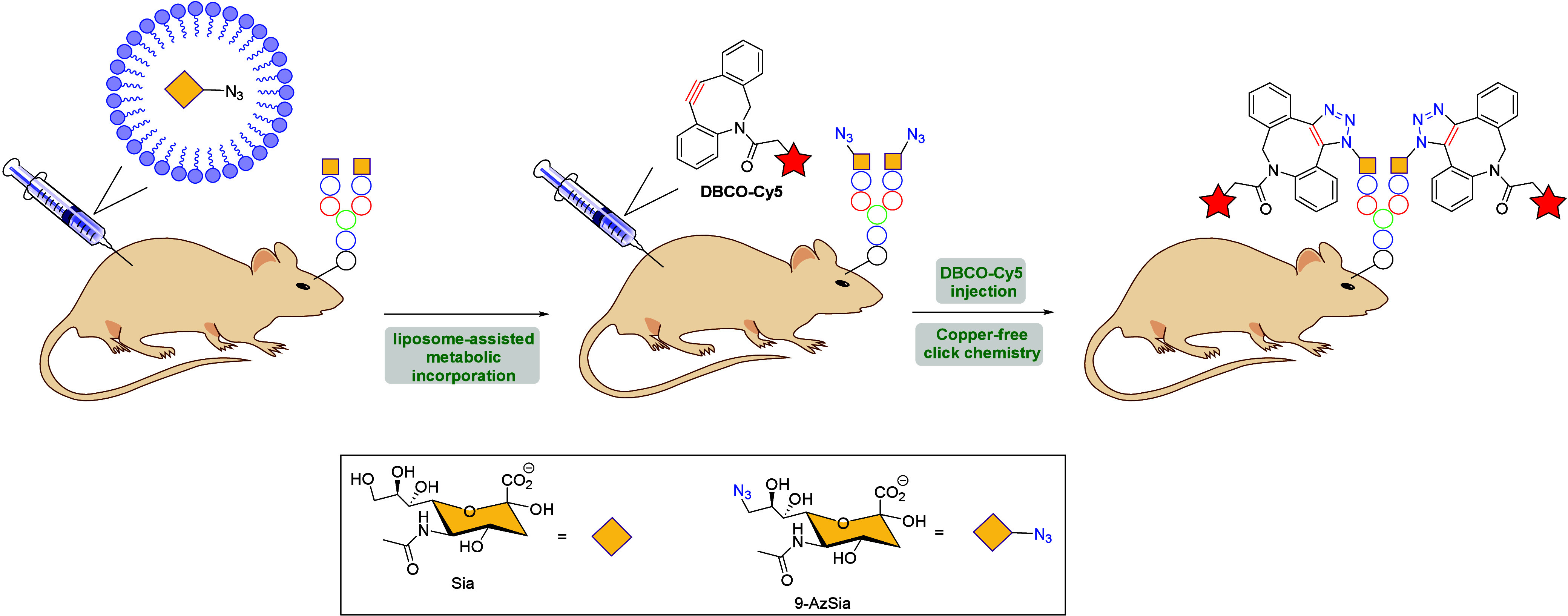
Liposome-Assisted Bioorthogonal Reporter (LABOR) Strategy *via* SPAAC in Living Mice

#### 3.2.3 Synthesis of Glycofullerenes and Glycovaccines via SPAAC Reaction

The SPAAC reaction has also been applied to the preparation of multivalent systems based on hexakis-adducts of [60]­fullerene bearing multiple carbohydrate units, which are crucial for biological recognition processes, where multivalent presentation is essential. Martín et al. were the first to describe the use of [60]­fullerene to prepare orthogonally nonsymmetric click-adducts containing both amino acid and monosaccharide units of biological relevance through a thiol-maleimide conjugation-SPAAC sequence (Scheme 26).^114^ This one-pot protocol allowed for the efficient preparation of mixed adducts that combined two different biomolecules.

**Scheme 26:**
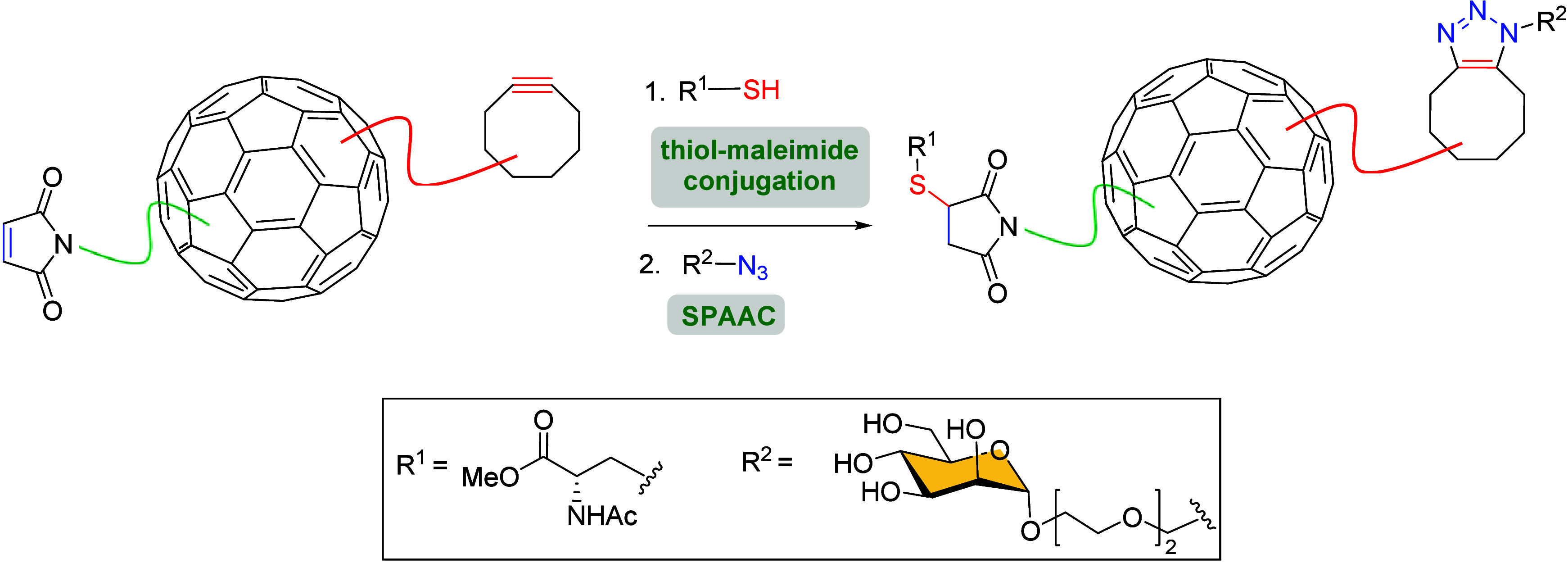
One-Pot Thiol-Maleimide Conjugation-SPAAC Sequence to Prepare Amino Acid-Monosaccharide [60]­Fullerene

The same authors advanced this concept by designing a series of antivirals using the SPAAC approach, targeting the blockade of carbohydrate receptors as a novel strategy to inhibit the viral infection process.^115,116^ They prepared a water-soluble tridecafullerene bearing 360 mannobioside units, which was tested to block DC-SIGN,^115^ a receptor involved in the entry of virus such as ZIKV and DENV into the cells. The results showed the best IC_50_ values reported to date for both viruses (67 pM for ZIKV and 35 pM for DENV). This strategy highlights the utility of the SPAAC reaction for the development of new antivirals against ZIKV, especially since there is currently no approved specific antiviral drug for treating of ZIKV infections.

Another interesting application of the SPAAC reaction in carbohydrate chemistry for therapeutic purposes is the preparation of carbohydrate-based vaccines. Adamo et al. reported a two-step protocol for the covalent conjugation of a polysaccharide antigen, functionalized with a cyclooctyne moiety, to a protein, which is conveniently modified with an azido-linker **76** (Scheme 27).^117^ Initial studies reacted small-medium-sized glycans bearing a monofluorinated cyclooctyne (MFCO) arm with predetermined tyrosine residues of the CRM_197_ carrier protein **77**, having an azide-linker via free-metal cycloaddition. The corresponding glycoconjugates vaccines were efficiently obtained with defined attachment points. Once the efficiency of this approach was proven, the same authors prepared a series of streptococcal polysaccharides bearing MFCO groups for the chemoselective conjugation to azide-containing Group B Streptococcus (GBS) pilus proteins as vaccine antigens, enabling the coupling of streptococcal polysaccharides. This technology provides an adequate strategy for selectively incorporating carbohydrates into proteins in the preparation of efficacious vaccines.

**Scheme 27:**
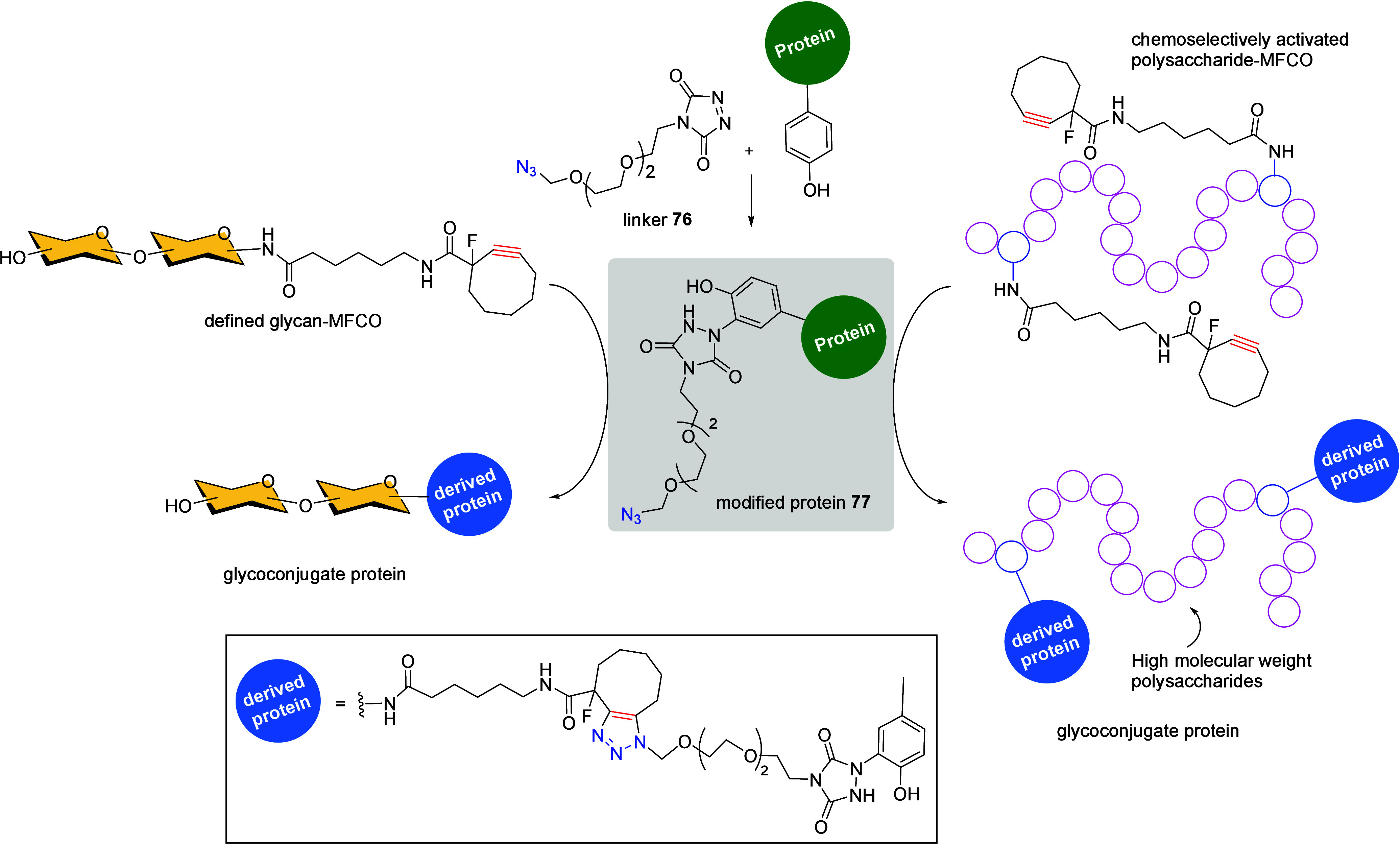
Tyrosine-Ligation SPAAC Reaction for the Synthesis of Glycoconjugates

#### 3.2.4 SPAAC Application for Developing Lysosome-Targeting Chimeras (LYTACs)

Targeted protein degradation (TPD) has emerged as a promising strategy for therapeutic development and a powerful tool in chemical biology. Among TPD approaches, proteolysis targeting chimeras (PROTACs) are notable for leveraging the ubiquitin-proteasome system (UPS) to selectively degrade intracellular proteins.^118^ This capability allows researchers to investigate biological pathways and cellular degradation mechanisms, particularly for cancer treatment.^119^ However, PROTACs are limited, because they cannot target extracellular proteins, restricting their therapeutic applications. Since extracellular and membrane proteins constitute about 40% of the proteome and play key roles in various diseases, lysosome-targeting chimeras (LYTACs) have been developed to address this gap. LYTACs, inspired by PROTACs, are cutting-edge bifunctional molecules that direct extracellular and membrane-bound proteins to lysosomes for degradation. They are composed of an antibody target protein binder and a lysosome-targeting receptor binder (Figure 2A). This strategy allows the selective breakdown of proteins that traditional UPS-based methods cannot reach, broadening the therapeutic potential of TPD to include diseases involving extracellular and membrane proteins.^120^

**Figure 2:**
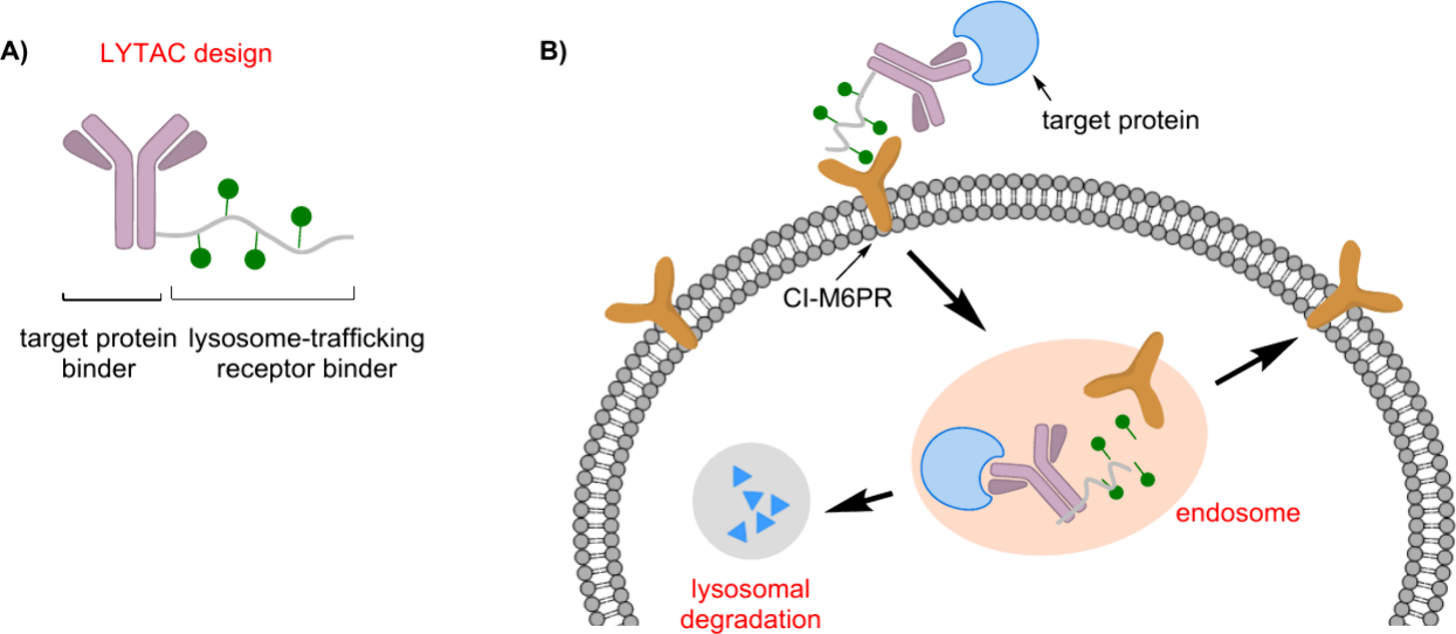
LYTACs use a glycopolypeptide ligand targeting CI-M6PR, conjugated to an antibody, to direct secreted and membrane-associated proteins to lysosomes.

Bertozzi’s group was the first to develop LYTACs using the SPAAC reaction, focusing on the cation-independent mannose-6-phosphate receptor (CI-M6PR). Their initial LYTACs linked a mannose-6-phosphate-based polyvalent ligand to an antibody that targets a specific protein for degradation. This complex then binds to CI-M6PR, resulting in the internalization of the complex and its delivery to the lysosome for degradation (Figure 2B).^121^

To enhance the multivalent presentation, they synthesized a glycopolypeptide (Poly­(M6Pn-co-Ala) with multiple serine-O-mannose-6-phosphonate (M6Pn) residues (Scheme 28). This process began with the conversion of mannose pentaacetate into *N*-carboxyanhydride (NCA)-derived glycopolypeptides (M6Pn-NCA) through a 13-step synthesis. Subsequent copolymerization of M6Pn-NCA and alanine-NCA resulted in an M6Pn glycopolypeptide (Poly­(M6Pn-co-Ala). To conjugate the Poly­(M6Pn)-bearing glycopolypeptide to an antibody, the authors labeled Poly­(M6Pn-co-Ala) with bicyclononyne to obtain Poly­(N6Pn-co-Ala)-BCN and then coupled it to the antibody (previously functionalized with an azide group) through SPAAC (Scheme 28). They first targeted EGFR protein, a known driver of cancer proliferation that functions beyond receptor tyrosine kinase activity inhibition.^122^ LYTACs were constructed using cetuximab (ctx), an FDA-approved EGFR-blocking antibody, and they were capable of effectively degrading EGFR protein.

**Scheme 28:**
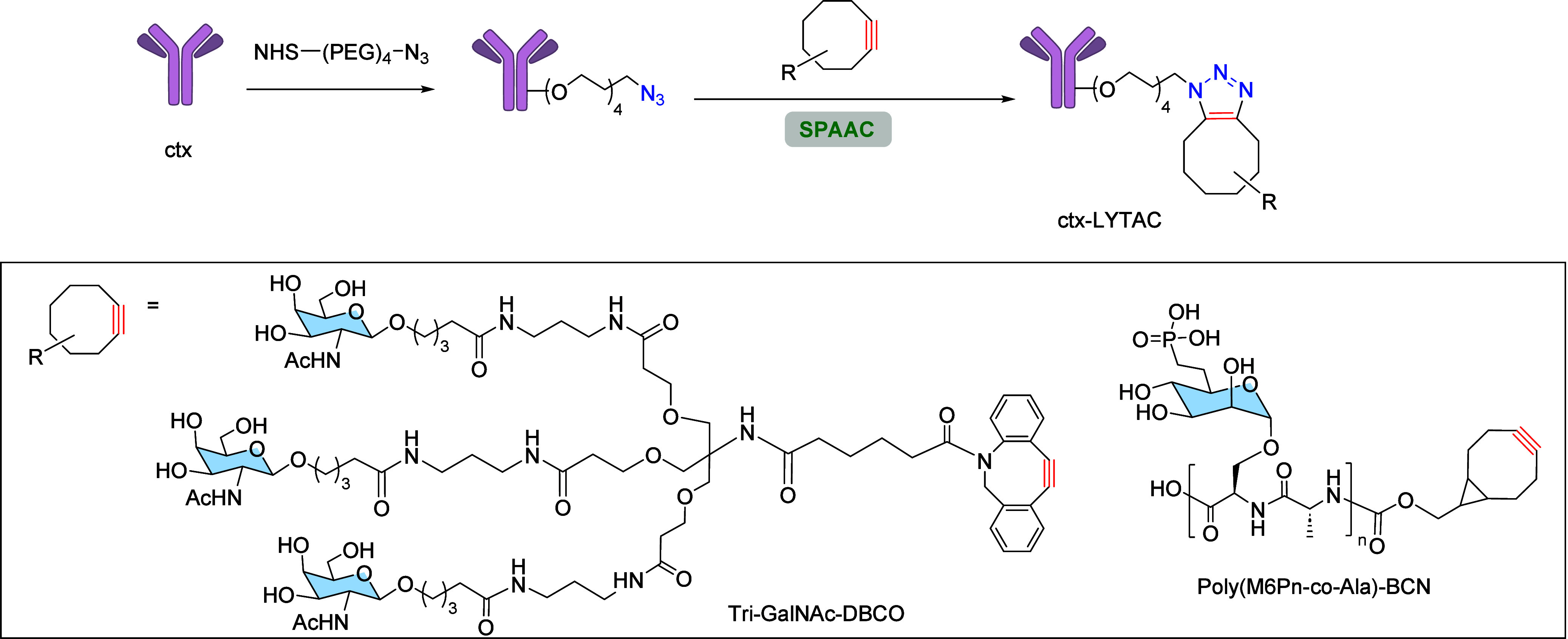
Synthesis of LYTACs Using SPAAC Reaction

Furthermore, the same group developed a second generation of LYTACs using the SPAAC reaction, targeting the liver-specific asialoglycoprotein receptor (ASGPR). ASGPR, a lectin expressed on liver cells, recognizes glycoproteins bearing *N*-acetylgalactosamine (GalNAc) or galactose ligands, internalizing them *via* endocytosis, followed by lysosomal degradation. Therefore, LYTACs using GalNAc ligands were developed to engage ASGPR, achieving higher internalization efficiency due to elevated expression of ASGPR in hepatocytes. Initially, triantennary GalNAc ligands (Tri-GalNAc-DBCO) were synthesized in 8 steps from peracetylated GalNAc and then conjugated to antibodies via SPAAC methodology (Scheme 28), demonstrating effective degradation of EGFR in hepatocellular carcinoma cell lines.^123^

This study underscores the potential of GalNAc-LYTACs for targeted protein degradation, particularly in treating diseases such as hepatocellular carcinoma with high specificity and minimal off-target effects. By utilizing liver-specific receptors like ASGPR, the therapeutic scope of LYTACs is expanded, paving the way for new treatments and insights into cellular biology.^124^

#### 3.2.5 SPAAC Combine with Biocatalysis for the Preparation of Glycoconjugates with Therapeutic Interest

The SPAAC reaction and biocatalysis have rapidly grown, driven by the development of robust biocatalysts and the widespread use of efficient click reactions. This convergence has given rise to “bioclick chemistry”, which combines biocatalytic enzyme activity with reliable click reactions for green and sustainable synthesis of high-value molecules.^125^ This section will highlight two examples of bioclick chemistry, focusing on applications in glycochemistry and sustainable synthesis.

In the first example van Delft et al.^126^ established a nongenetic technology termed GlycoConnect based on the conversion of native monoclonal antibodies (mAbs) into Antibody–Drug Conjugates (ADCs) through a combination of enzymatic synthesis with a SPAAC (Scheme 29). This three-step protocol involves: (i) trimming a native mAb **78**, which is a mixture of glycoforms at Asn-297, with an endoglycosidase to cleave the GlcNAc–GlcNAc linkage, resulting in structure **79**; (ii) the catalytic attachment of an azide-modified GalNAc moiety using a glycosyl transferase to generate **80**; and (iii) anchoring the bicyclononyne-modified toxic payload component (trastuzumab and maytansine) via the SPAAC reaction, yielding the corresponding ADC **81** with high stability and homogeneity. This GlycoConnect technology is very promising as a targeted therapy with a superior therapeutic index.

**Scheme 29:**
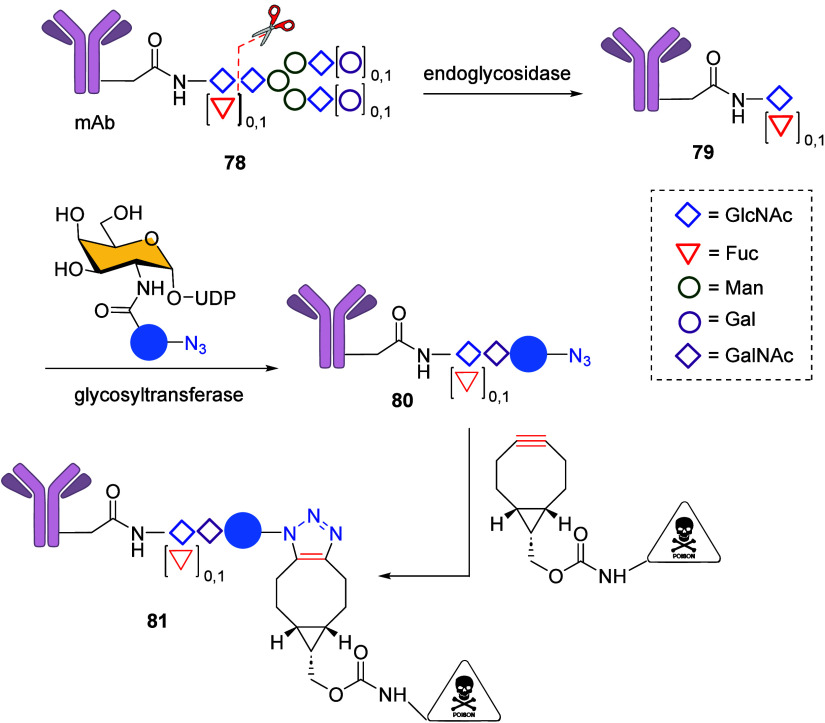
Development of GlycoConnect Based on Bioclick Chemistry Combining Biocatalytic Synthesis with SPAAC Reaction^a^

a UDP = uridine diphosphate.

Another example of bioclick chemistry was reported by Bojarová et al., who developed biocompatible glyconanomaterials based on N-(2-hydroxypropyl)­methacrylamide (HPMA) copolymers for the specific targeting of galectin-3 (Gal-3). This protein is a promising target in cancer therapy because it is abundantly localized in tumor tissue and plays a crucial role in tumor development and proliferation.^127^ However, the clinical application of Gal-3-targeted inhibitors is often challenged by issues of insufficient selectivity and low biocompatibility. The authors envisioned HPMA-based nanocarriers pending Gal-3-targeted inhibitors as attractive glyconanomaterials for in vivo applications due to their good water solubility, low toxicity, and lack of immunogenicity. The enzymatic synthesis of a specific functionalized GalNAcβ1 → 4GlcNAc (LacdiNAc) epitope pending an azide group was accomplished by mutant β-N-acetylhexosaminidases (Scheme 30A). Then, the biocompatible HPMA copolymer decorated with cyclooctyne functionalities was combined with the Gal-3 specific epitope LacdiNAc by SPAAC to effectively target Gal-3 (Scheme 30B).

**Scheme 30:**
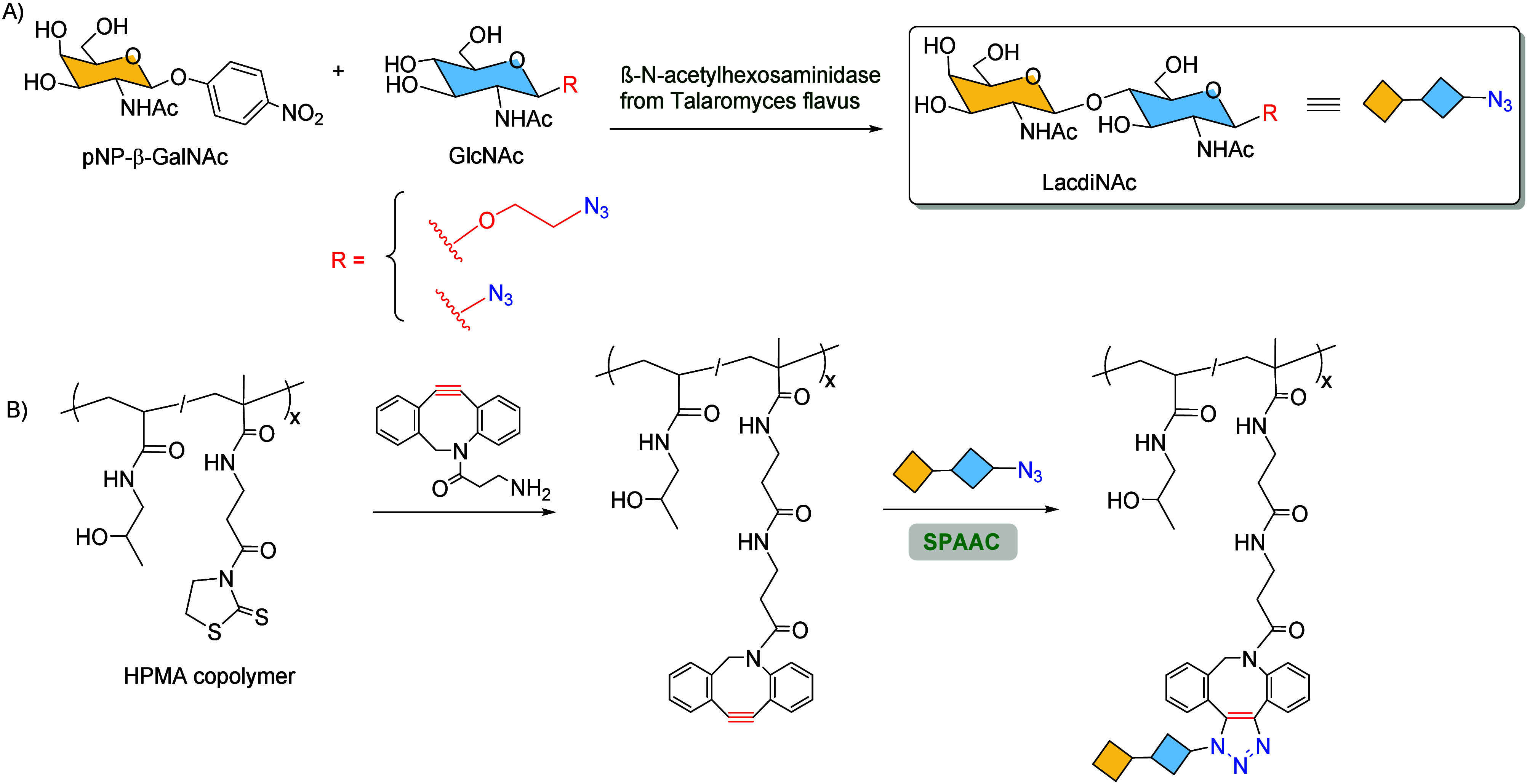
Development of Glycomaterials Based on Bioclick Chemistry

In summary, the SPAAC, first explored by Bertozzi and later by other researchers, has emerged as the best candidate for the bioorthogonal functionalization of biomolecules with probes in biological systems and living organisms, due to its extreme selectivity, biocompatibility, and fast rate kinetics. The SPAAC reaction has established itself as a powerful tool for bioconjugation, enabling the *in vivo* visualization of glycan dynamics in various biological and pathological processes. Moreover, the remarkable properties of SPAAC have allowed its applications in fields such as biomedicine, material science, bioengineering, and nanotechnology, among others. For this reason, its potential is still being explored, promising further advancements and applications in all of these areas.

## 4 Inverse Electron-Demand Diels–Alder (IEDDA) Reaction

It is also worth mentioning the inverse electron-demand Diels–Alder (IEDDA) reaction as another prominent example of a biorthogonal ligation method.^39^ First introduced in 2008 by Blackman et al.^40^ has become widely recognized for its versatility across various fields of chemistry and chemical biology.^41^ This reaction, based on the classical Diels–Alder cycloaddition mechanism, has gained prominence due to its ability to selectively and efficiently couple functional groups in complex biological environments. Originally developed to overcome the limitations of traditional Diels–Alder reactions in biological systems, the IEDDA reaction has recently emerged as a powerful tool in carbohydrate modification. It involves a rapid and selective cycloaddition between an electron-deficient diene (such as a tetrazine) and an electron-rich dienophile (such as a strained alkene). This reaction proceeds under mild conditions with exceptional properties, making it well suited for applications in glycoscience (Table 1).

One of the most relevant applications of the IEDDA reaction is in metabolic glycoengineering, which is a powerful approach for modifying cell surface glycans to study their biological functions, track glycan dynamics, and develop glycan-based therapeutic approaches. A major challenge in this field is the need for highly selective and rapid bioorthogonal reactions that enable the efficient labeling and functionalization of glycans in live cells. The IEDDA reaction meets these demands due to its exceptional kinetic efficiency, biocompatibility, and specificity. It proceeds without interference from native biomolecules, making it ideal for glycan labeling, live-cell imaging, and targeted drug delivery.^128^ The first example was reported in 2012 by the Prescher group,^129^ involving a sialic acid modified with a methyl-substituted cyclopropene (9-Cp-NeuAc). This derivative was metabolically incorporated into glycans on the surface of Jurkat cells and labeled through a two-step process using tetrazine-biotin, followed by an avidin-dye conjugate. Incorporation efficiency was assessed via flow cytometry (Scheme 31). Additionally, some cyclopropene moieties were integrated into cell surface structures and subsequently detected by using covalent probes. Due to their small size and high selectivity, cyclopropenes hold great potential for tagging a wide range of biomolecules *in vivo*.

**Scheme 31:**
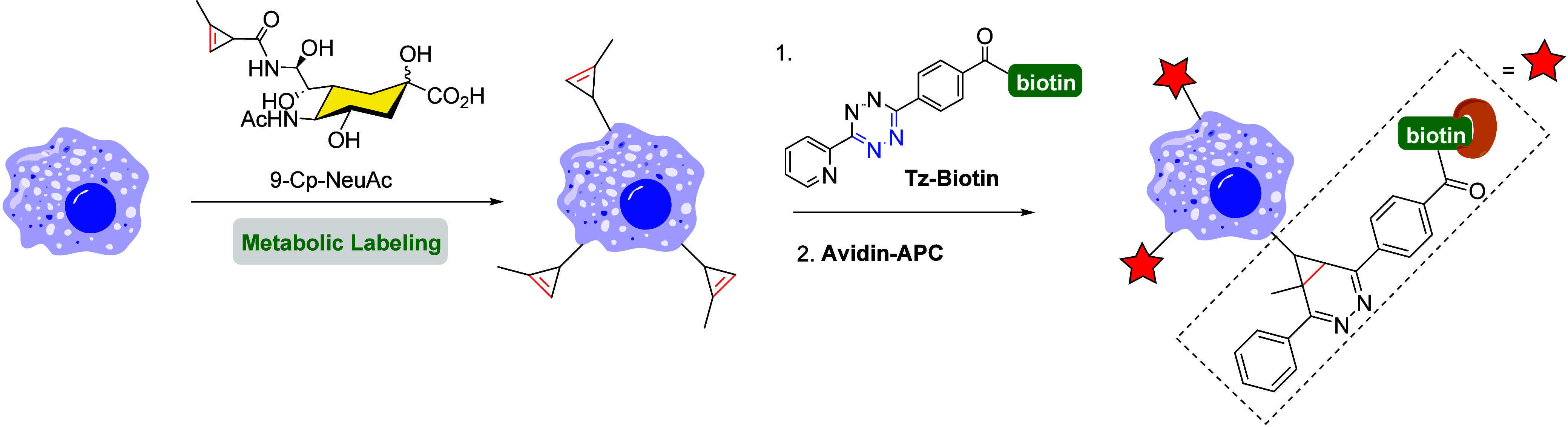
Cyclopropene-Modified Sialic Acid (9-Cp-NeuAc) Can Be Metabolically Incorporated onto Live Cell Surfaces Using IEDDA Reaction

Another remarkable application of the IEDDA reaction is the development of injectable polysaccharide-based hydrogels. These materials have attracted significant attention in biomedical research because of their biocompatibility, biodegradability, and minimally invasive therapeutic interventions. Several polysaccharides, such as hyaluronic acid, alginate, chitosan, cellulose, and heparin, have been functionalized with the telechelic groups required for the IEDDA reaction. These modifications, which introduce dienes (primarily tetrazines) and dienophiles (such as norbornenes and *trans*-cyclooctenes) at low degrees of substitution, preserve the water solubility of the polysaccharides. When aqueous solutions of tetrazine-modified polysaccharides interact with dienophile-functionalized counterparts, rapid gelation occurs within minutes.^130^

## 5 Sulfur(VI) Fluoride Exchange Reaction (SuFEx)

### 5.1 Introduction

Despite SPAAC and TEC being the main metal-free click reactions, this concept extends to other processes, including hetero-Diels–Alder cycloaddition, thiol-Michael addition and oxime ligation, among others.^131^ Recently, sulfur­(VI) fluorine exchange (SuFEx) has emerged as a novel type of click reaction,^42^ although it was identified long ago. This reaction involves sulfonyl fluorides or fluorosulfates reacting with various nucleophiles to effectively form R-SO_2_–Y/RO–SO_2_–Y (Y = N, O) type linkages. Special focus has been given to sulfonamide group formation, as it is a crucial structural motif found in multiple therapeutic agents.^132,133^

The formation of S–O bonds via SuFEx coupling is achieved from the corresponding sulfonyl fluorides or fluorosulfates using silyl ethers as nucleophiles in the presence of a non-nucleophilic base. The formation of the strong Si–F bond in the silyl fluoride byproduct drives the reaction. Similarly, the sulfonamide bond is successfully prepared by reacting sulfonyl fluoride with an excess of amine to neutralize the HF released during the reaction.

This novel reaction offers an alternative to the common nucleophilic addition of C-, N-, and O-nucleophiles to highly reactive sulfonyl chlorides, which are quite unstable under reductive and basic conditions. The success of SuFEx chemistry relies on the exceptional properties of sulfonyl fluorides, including: (i) ease of preparation, (ii) inertness to oxygen and water, (iii) hydrolytic stability under acidic and basic conditions, and (iv) high and selective-S reactivity toward C-, N- and O-nucleophiles.^42^

Therefore, this reaction provides rapid and easy access to the S–O and S–N bonds under mild conditions in a highly effective manner. It has been demonstrated to be orthogonal to other click reactions, such as SPAAC and TEC.^134^ Moreover, the efficiency and the potential of this methodology have been validated in polymer chemistry and materials science. It also appears to be suitable for conjugation in biological systems, where other click reactions cannot be applied. Besides, the potential of this methodology has been recently expanded to carbohydrate chemistry.

### 5.2 Applications

The application of SuFEx in carbohydrates to connect a sugar ring to a scaffold via −SO_2_–N bond formation was first reported by Dondoni and co-workers in 2016.^43^ Their studies focused on the synthesis of carbohydrate sulfonamides to develop new molecules with potential clinical applications, including antibacterial diuretics, anticonvulsants, and HIV protease inhibitors.^132,133^ Accordingly, *C*-glucosylsulfonyl fluoride **82a** was reacted with various amines in DMF at 80 °C in the presence of an excess of base to neutralize the HF released during sulfonamide bond formation, affording *C*-glucosylsulfonyl amides **84a**–**e** in good to excellent yields (Scheme 32). Under these optimal conditions, glucosylsulfonyl fluoride **82a** was reactive toward primary and secondary alkyl amines, but it was inert toward arylamines, even under forcing conditions. This chemoselectivity highlights SuFEx as a selective method for preparing *N*-alkyl sulfonyl amides.

**Scheme 32:**
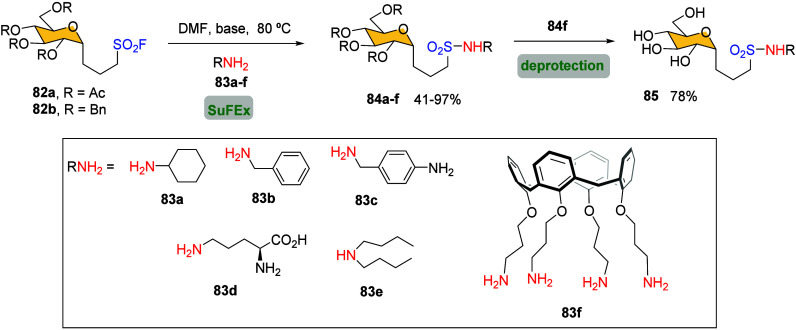
Synthesis of Aliphatic Sulfonamides **84**

The reaction scope was extended to obtain multivalent carbohydrate architectures, which are known to be involved in many biological recognition phenomena by interacting with proteins located on cell-membrane surface.^135^ Hence, Dondoni et al. successfully prepared the tetravalent glucosylsulfonamido calix[4]­arene **85** by conjugation of calix[4]­arene derivative **83f** with glucosylsulfonyl fluoride **82b** under previous conditions after an additional deprotection step (Scheme 32).^43,136^ The variation of the protecting group in the sugar sulfonylating agent from an O-acetyl to an O-benzyl group was due to the preferential transfer of the acetyl group from the sugar ring to the amino groups in cluster **83f** under reaction conditions.

Encouraged by these results, the same group attempted to attach different sulfonyl fluoride-derived sugar rings to the central cluster core using this two-step methodology. Specifically, they tried to synthesize calix[4]­arenes pending iminosugars motifs, but the corresponding iminosugar sulfonyl fluoride decomposed under reaction conditions.^43^ To overcome this limitation, the authors envisioned a complementary approach to achieve isomeric sulfonamide bioisosters. They accomplished the reaction between the amino group attached to the iminosugar ring and sulfonyl fluoride tethered to the cluster motif, yielding the sugar sulfonamide cluster in good overall yields after the debenzylation reaction.

In summary, while the SuFEx reaction is still in its early stages and requires further investigation, it seems to be an efficient, accessible, and complementary ligation tool that can complement other click reactions like TEC and strain promoted azide–alkyne cycloaddition. Furthermore, previous studies have shown that SuFEx is a viable metal-free method for the functionalization of proteins. This underscores their potential as a promising area for future research in the preparation of glycoconjugates in biological systems.

## 6 Conclusions and Perspectives

The development of efficient and biocompatible click chemistry reactions has significantly advanced the fields of glycochemistry and glycobiology. Metal-free click reactions, including TEC, SPAAC, IEDDA and SuFEx, have enabled the synthesis of complex glycoconjugates under mild conditions with exceptional regio- and stereoselectivity. These methodologies have opened new avenues in therapeutic design, diagnostics, materials science, and bioengineering by providing versatile, sustainable, and high-yielding approaches to functionalize biomolecules.

The TEC reaction represents a pivotal advancement in glycochemistry, providing an efficient, regio- and stereoselective approach for synthesizing complex glycoconjugates under mild conditions. TEC’s adaptability has facilitated its application in diverse areas, including the synthesis of *S*-polysaccharides with enhanced stability, sugar-modified nucleosides with improved therapeutic properties, and structurally defined glycopeptides and glycoproteins. Its integration into glycoconjugate vaccine development and the design of glycosylated biomaterials underscore its potential to address critical challenges in drug discovery, vaccine formulation, and biomaterial engineering. Future efforts should prioritize optimizing TEC for large-scale applications, exploring its compatibility with complementary bioconjugation techniques, and expanding its use in synthesizing novel glycomimetics and bioactive compounds.

The SPAAC has emerged as a transformative bioorthogonal reaction, providing a nontoxic, rapid, and selective tool for biomolecule labeling and imaging. Its ability to operate under physiological conditions makes it invaluable for studying glycosylation dynamics, metabolic pathways, and disease-related biomarkers in complex biological systems. Innovations such as structurally optimized cyclooctynes and advanced metabolic precursors have further enhanced its reactivity and versatility. The successful application of SPAAC in human tissues and live animals highlights its potential for translational research and diagnostic development. Future efforts should focus on overcoming existing challenges to fully harness SPAAC’s capabilities in advancing glycoscience and biomedicine.

SPAAC reaction has also revolutionized glycoconjugate synthesis, including the preparation of glycofullerenes for antiviral strategies, glycovaccines targeting infectious diseases, and lysosome-targeting chimeras (LYTACs) for selective protein degradation. Additionally, SPAAC’s integration with biocatalysis demonstrates its potential for sustainable synthesis, exemplified by the development of antibody–drug conjugates (ADCs) and glyconanomaterials for targeted cancer therapies. Beyond its biomedical impact, SPAAC has driven innovations in materials science and bioengineering, positioning itself as a cornerstone technology for therapeutic and diagnostic advancements in glycobiology.

The IEDDA reaction is a highly efficient and selective metal-free cycloaddition that has become a valuable tool in chemical biology due to its rapid kinetics, biocompatibility, and mild aqueous conditions. In glycoscience, it enables selective labeling of cell-surface glycans in living cells, making it ideal for live-cell imaging, glycan tracking, and therapeutic development. Its bioorthogonality allows for in vivo applications without disrupting native biomolecules. IEDDA has also been used to create injectable hydrogels by functionalizing natural polysaccharides (e.g., hyaluronic acid, alginate, and chitosan) with tetrazines and strained dienophiles, enabling rapid gelation for biomedical uses such as drug delivery and tissue engineering.

The SuFEx reaction has emerged as a powerful tool in metal-free click chemistry, offering a unique combination of efficiency, chemoselectivity, and stability under mild conditions. Its ability to form sulfonamide and sulfonate bonds with high specificity and compatibility has been demonstrated in diverse applications, including the synthesis of bioactive carbohydrate sulfonamides and multivalent glycosylated architectures. While still in its early stages, SuFEx complements existing click reactions, broadening the toolbox for glycoconjugate synthesis. The reaction’s orthogonality and utility in functionalizing proteins suggest potential applications in biological systems where other click reactions face limitations. Future research focused on expanding its substrate scope and optimizing conditions will further enhance its role as a transformative tool in chemical biology, therapeutic development, and materials science.
